# Supplementary material for: Tailored interventions into broad attitude networks towards the COVID-19 pandemic
Source: PLoS One. 2022 Oct 27;17(10):e0276439. doi: 10.1371/journal.pone.0276439 (PMC9612523; doi:10.1371/journal.pone.0276439)
Supplement: S1 File — (PDF) [file pone.0276439.s001.pdf]

Supplement

Tailored interventions into broad attitude networks towards the COVID-19 pandemic

Contents

Contents 1
1. Additional information on procedure 2
1.1 Psychological determinants of compliance during pandemics 2
1.2 Survey items per node in the COVID-19 broad attitude network 4
1.3 Interventions 9
1.3.1 Interventions T3 9
1.3.1.1 Trust 9
1.3.1.2 Social Norm 13
1.3.1.3 T3 control condition 17
1.3.2 Interventions T5 18
1.3.2.1 Measures Support 18
1.3.2.2 Economic Consequences 22
1.3.2.3 T5 control condition 26
2. Additional information on analyses 28
2.1 Dropped out participants longitudinal study 28
2.2 Node construction and Principal Axis Factoring 29
2.3 Detailed description per node 35
2.4 R script 40
2.5 Additional information on Network Analysis 40
3. Additional information on results 41
3.1 Part 1 - Network structure 41
3.1.1 Detailed descriptive account of COVID-19 broad attitude network 41
3.1.2 Community stability and detection analysis 47
3.1.3 Network without threshold 49
3.1.4 Edge weights 50
3.1.5 Node strength 51
3.1.6 Edge accuracy, Edge difference, Centrality stability, and Centrality difference 52
3.2 Part 2 - Interventions 55
3.2.1 Descriptives intervention conditions 55
3.2.2 Significance values of difference tests per node 57
4. References 58

1. Additional information on procedure

1.1 Psychological determinants of compliance during pandemics

The psychological variables included in the survey are based on an extensive literature review of health behavior during a pandemic by Bish and Michie [1]. Their proposed frameworks, with determinants depending on types of protective behaviors, are covered by several models, and complemented with other relevant variables. Prominent generic models within (health) psychology contributing to explaining health behavior are, among others, the Theory of Planned Behavior (TPB) and the Health Belief Model (HBM). The TPB [2] emphasizes the importance of attitudes, behavioral control and the subjective norm on behavior. The HBM [3, 4] focusses on perceived susceptibility and severity of the disease, benefits and barriers of behavior, self-efficacy and cues to action to explain health-related behavior. Studies into compliance with behavioral measures in the context of pandemics confirm the importance of the factors in these models, but also identify additional factors to play a role. More specifically, recent research into the COVID-19 pandemic associated multiple factors from the TPB and HMB with compliance, such as attitudes [5], social norms [6], self-efficacy [7], perceived controllability and severity [8], and risk perception [9]. Factors that contribute to explaining behavior during pandemics in addition to those in TPB and HMB are for instance trust in authorities and science [1, 9, 10], knowledge [11, 12] and demographics and personality variables [13, 14]. This underlines the relevance of including a broad range of psychological factors extending beyond those in the prevailing models of (health) behavior to explain compliance with behavioral measures during this unprecedented and complex COVID-19 pandemic.

Taking into account the effects of the pandemic on the public's well-being is important. For instance, social isolation and loneliness, both potential consequences of the behavioral measures during the COVID-19 pandemic, are associated with adverse mental and

physical health effects [15]. Previous research also found associations between anxiety and preventive behaviors during pandemics [1]. More specifically, fear as a result from the pandemic is related to compliance with behavioral measures during the COVID-19 pandemic [13, 16], which further emphasizes the importance of taking into account (changes in) well-being during pandemics.

In conclusion, understanding the complex interplay of psychological factors underlying compliance with behavioral measures during the COVID-19 pandemic requires an integral and systemic approach, encompassing a broad range of variables relevant to behavioral compliance as well as factors relating to mental and physical health.

1.2 Survey items per node in the COVID-19 broad attitude network

Table S1. Survey items per node

Node	Survey items - (R) = reverse score	Answer scale
**Compliance T1**	I comply with the corona measures.	1 (*I never comply with the measures*) to 7 (*I always comply with the measures*)
	Stay at home as much as possible.	1 (*I do not display this behavior more*) to 7 (*I display this behavior much more now*)
	Keep 1.5 meters away from others.	
	Wash your hands regularly with soap and water.	
	Cough and sneeze into the inside of your elbow.	
**Compliance T2**	I comply with the corona measures.	1 (*I never comply with the measures*) to 7 (*I always comply with the measures*)
	Keep 1.5 meters away from others.	1 (*I do not display this behavior more*) to 7 (*I display this behavior much more now*)
	Wash your hands regularly with soap and water.	
	Cough and sneeze into the inside of your elbow.	
	Avoid crowds.	
**Compliance T3 -T5**	I comply with the corona measures.	1 (*I never comply with the measures*) to 7 (*I always comply with the measures*)
	Keep 1.5 meters away from others.	1 (*I do not display this behavior more*) to 7 (*I display this behavior much more now*)
	Wash your hands regularly with soap and water.	
	Cough and sneeze into the inside of your elbow.	
	Avoid crowds.	
	Stay close to home.	
**Risk Perception**	How likely do you think it is that u will get infected with the coronavirus in the upcoming year?	1 (*Extremely unlikely*) to 7 (*Extremely likely*)
	How severe do you think an infection with the coronavirus would be for you?	1 (*Not severe*) to 7 (*Very severe*)
**Health Risk**	For me personally, I consider the health risk of an infection with the coronavirus..	1 (*Extremely small*) to 7 (*Extremely severe*)
	For my family and friends, I consider the health risk of an infection with the coronavirus..	
**Economic Consequences**	For me personally, I consider the economic consequences of the corona pandemic ..	1 (*Extremely small*) to 7 (*Extremely severe*)
	For my family and friends, I consider the economic consequences of the corona pandemic are..	
**Self-exempting Beliefs**	I will not get infected with the coronavirus because I never get the seasonal flu (influenza) either.	1 (*Strongly disagree*) to 7 (*Strongly agree*)
	I think I am already immune (protected) against the coronavirus.	
**Negative Affect**	The corona pandemic is making me angry.	1 (*Strongly disagree*) to 7 (*Strongly agree*)
	The corona pandemic is making me sad.	

	The corona pandemic is making me feel confused.	
	The corona pandemic is making me feel uncertain.	
	The corona pandemic is making me feel overwhelmed.	
	The corona pandemic is making me feel frustrated.	
	The corona pandemic is making me fearful.	
	The corona pandemic is making me feel out of control.	
**Compassion**	The corona pandemic is making me feel compassion.	1 (*Strongly disagree*) to 7 (*Strongly agree*)
**Worries Virus**	I worry about getting infected with the coronavirus.	1 (*Do not worry at all*) to 7 (*Worry a lot*)
	I worry about infecting others with the coronavirus.	
	I worry about losing someone I love.	
	I worry about the health care system overloading.	
**Worries Measures**	I worry about what staying at home a lot will do to my health.	1 (*Do not worry at all*) to 7 (*Worry a lot*)
	I worry about the schools closing.	
	I worry about a recession.	
	I worry about limited access to food.	
	I worry about losing my job.	
	I worry about getting lonely.	
**Vaccination Intention**	If a vaccine becomes available, I would get it.	1 (*Strongly disagree*) to 7 (*Strongly agree*)
**Measures Support**	I find the corona measures...	1 (*Senseless*) to 7 (*Sensible*)
	I find the corona measures...	1 (*Useless*) to 7 (*Useful*)
	I find the corona measures...	1 (*Unnecessary*) to 7 (*Necessary*)
	I find the corona measures...	1 (*Unfair*) to 7 (*Fair*)
	I find the corona measures...	1 (*Unacceptable*) to 7 (*Acceptable*)
	I think the corona measures will prevent the spread of the coronavirus.	1 (*Strongly disagree*) to 7 (*Strongly agree*)
	As a society, we must do whatever is necessary to prevent the spread of the coronavirus.	
**Measures Ease**	I find the corona measures...	1 (*Unpleasant*) to 7 (*Pleasant*)
	I find the corona measures...	1 (*Difficult*) to 7 (*Easy*)
**Social norm**	I think the majority of people comply with the corona measures.	1 (*Strongly disagree*) to 7 (*Strongly agree*)
	I think majority of people find it important that people comply with the corona measures.	
**Control Infection**	For me personally, avoiding an infection with the coronavirus in the current situation is…	1 (*Extremely* difficult) to 7 (*Extremely* easy)
	For my family and friends, avoiding an infection with the coronavirus in the current situation is…	
**Self-efficacy**	I know how to protect myself from the coronavirus.	1 (*Strongly disagree*) to 7 (*Strongly agree*)
**Involvement**	How important is the topic of corona pandemic to you?	1 (*Very unimportant*) to 7 (*Very important*)
	To what extent does the news about the corona pandemic have your attention?	1 (*Not at all*) to 7 (*Very much)*

	How much do you think about the corona pandemic?	1 (*Very little*) to 7 (*Very much*)
**Perceived Knowledge**	How much knowledge do you think you have about the corona pandemic?	1 (*Very little*) to 7 (*Very much*)
**Trust**	I trust the authorities to adequately manage the corona pandemic.	1 (*Strongly disagree*) to 7 (*Strongly agree*)
	I trust RIVM during the corona pandemic.	
	I trust health care professionals during the corona pandemic.	
	I trust science during the corona pandemic.	
**Consideration of future consequences**	Often I engage in particular behavior in order to achieve outcomes that may not result for many years. (R)	1 (*Strongly disagree*) to 7 (*Strongly agree*)
	My convenience is a big factor in the decisions I make or the actions I take. (R)	
	I am willing to sacrifice my immediate happiness or well-being in order to achieve future outcomes.	
	I think it is important to take warnings about negative outcomes seriously even if the negative outcome will not occur for many years.	
	I generally ignore warnings about possible future problems because I think the problems will be resolved before they reach crisis level. (R)	
**Resilience**	I tend to bounce back quickly after hard times.	1 (*Strongly disagree*) to 5 (*Strongly agree*)
	I have a hard time making it through stressful events. (R)	
	It does not take me long to recover from a stressful event.	
	It is hard for me to snap back when something bad happens. (R)	
	I usually come through difficult times with little trouble.	
	I tend to take a long time to get over set-backs in my life. (R)	
**Coping**	I think that I have to accept that this has happened.	1 ([*almost] Never*) to 5 ([*almost] Always*)
	I think of something nice instead of what has happened.	
	I think that I can become a stronger person as a result of what has happened.	
	I think that I have to accept the situation.	
	I think about how to change the situation.	
	I think I can learn something from the situation.	
	I think that it hasn't been too bad compared to other things.	
	I think of pleasant things that have nothing to do with it.	
	I think about a plan of what I can do best.	
	I tell myself that there are worse things in life.	
**Health General**	In general, how would you rate your health?	1 (*Very poor*) to 7 *(Very good)*
**Health Change Physical**	How would you rate your physical health now as compared to before the corona pandemic?	-3 (*Much worse*) to 3 (*Much better*)
**Health Change Mental**	How would you rate your mental health now as compared to before the corona pandemic?	-3 (*Much worse*) to 3 (*Much better*)
**Healthy Lifestyle**	I've been eating in the past two weeks, compared to before the corona pandemic...	-3 (*Much less healthy*) to 3 (*Much healthier*)
	I've been exercising in the past two weeks, compared to before the corona pandemic…	-3 (*Much less*) to 3 (*Much more*)

	I've been sleeping for the past two weeks, compared to before the corona pandemic...	-3 (*Much worse*) to 3 (*Much better*)
**Mental Wellbeing**	I’ve been feeling optimistic about the future.	1 (*Never*) to 5 (*Always*)
	I’ve been feeling useful.	
	I’ve been feeling relaxed.	
	I’ve been dealing with problems well.	
	I’ve been thinking clearly.	
	I’ve been feeling close to other people.	
	I’ve been able to make up my own mind about things.	
**Loneliness**	I experience a general sense of emptiness.	1 (*Not at all*) to 5 (*Very much*)
	There are plenty of people I can lean on when I have problems. (R)	
	There are many people I can trust completely. (R)	
	I miss having people around me.	
	There are enough people I feel close to. (R)	
	I often feel rejected.	
**Somatic Complaints**	To what extent did you experience dizziness or faintness during the past two weeks?	1 (*Not at all*) to 5 (*Very much*)
	To what extent did you experience chest pain during the past two weeks?	
	To what extent did you experience nausea during the past two weeks?	
	To what extent did you experience difficulty breathing during the past two weeks?	
	To what extent did you experience numbness during the past two weeks?	
	To what extent did you experience feeling weak during the past two weeks?	
**Depressive Complaints**	To what extent did you experience a loss of the will to live during the past two weeks?	1 (*Not at all*) to 5 (*Very much*)
	To what extent did you experience feeling lonely during the past two weeks?	
	To what extent did you experience feeling blue during the past two weeks?	
	To what extent did you experience a loss of interest in things during the past two weeks?	
	To what extent did you experience feeling hopeless about the future during the past two weeks?	
	To what extent did you experience a feeling of worthlessness during the past two weeks?	
**Anxiety Complaints**	To what extent did you experience nervousness during the past two weeks?	1 (*Not at all*) to 5 (*Very much*)
	To what extent did you experience feeling suddenly scared during the past two weeks?	
	To what extent did you experience feeling fearful during the past two weeks?	
	To what extent did you experience feeling tense during the past two weeks?	
	To what extent did you experience anxiety or panic attacks during the past two weeks?	
	To what extent did you experience restlessness during the past two weeks?	
**Age**	How old are you?	Open numeric field

**Gender**	What is your gender?	0 (*Male*) to 1 (*Female*)
**Education**	What is your highest level of education?	0 (*Primary/secondary*) to 1 (*Higher*) / n/a (*I prefer not to answer)*
**Smoking**	Do you smoke?	0 (*No*) to 1 (*Yes*) / n/a (*I prefer not to answer)*
**Illness**	Do you suffer from one or more of the following conditions?	0 (*No*) to 1 (*Yes*) / n/a (*I prefer not to answer)*
	Diabetes.<br>Hypertension.<br>Cancer.<br>Chronic (long-term) respiratory diseases, such as asthma, chronic obstructive pulmonary disease (COPD), emphysema or bronchitis.<br>Heart disease.<br>Chronic kidney disease.<br>Chronic liver disease, such as hepatitis.<br>Chronic neurological conditions, such as Parkinson's disease, motor neurone disease, multiple sclerosis (MS), a learning disability or cerebral palsy diabetes.<br>Problems with your spleen – for example, sickle cell disease or if you have had your spleen removed.<br>A weakened immune system as the result of conditions such as HIV and AIDS, or medicines such as steroid tablets or chemotherapy.<br>Seriously overweight (a BMI of 40 or above).	

1.3 Interventions

Original interventions are in Dutch. The webpage to the news article heading is provided in its caption.

1.3.1 Interventions T3

1.3.1.1 Trust

Trust low condition

Before we continue to the third survey, we would like to share a couple of news articles with you.

Poll: fewer people are positive about the government's corona approach

Picture of personal protective equipment (face mask, disinfectant and gloves)

Trust in that the government can properly manage the corona outbreak has decreased. The number of people who have that confidence has fluctuated around 70 percent for a long time, but has now decreased to 63 percent. This is the conclusion of consultancy firm Citisens after a poll among more than 2,500 people from its own research panel.

Source: https://nieuws.nl/algemeen/20200512/peiling-minder-mensen-positief-over-corona-aanpak-overheid/

Criticism of Prime Minister Rutte's corona policy comes from both left and right wing parties

Picture of Dutch politicians during a debate

The cabinet's corona strategy is receiving increasing criticism from the House of Representatives. Although left and right want to make adjustments in a completely different manner.

Source: https://www.trouw.nl/politiek/de-kritiek-op-het-coronabeleid-van-rutte-komt-van-links-en-rechts~bb7aab97/

The current study

The current study also shows that trust in the responsible authorities and experts has decreased. This could mean that the events and changes in policy of the past two weeks have a negative effect on people’s trust in the authorities, healthcare and science.

Explanation

The decrease of trust is due to the fact that **the responsible authorities and experts think differently about how to manage the pandemic** and disagree on key points. **The rules are also perceived as unclear and sometimes contradictory.** It seems that the authorities have little understanding of how the virus behaves and how it can be effectively managed.

To know if you have seen the information above, we ask you a question about this: According to the information above, has the level of trust in the responsible authorities and experts increased, decreased or remained the same?

1. Increased

2. Decreased
3. Remained the same

Trust high condition

Before we continue to the third survey, we would like to share a couple of news articles with you.

Research shows that the Dutch have confidence in the approach to the coronavirus

Picture of Jaap van Dissel, chairman of the Dutch Outbreak Management Team (photo adjusted)

The Dutch have confidence in the approach to the coronavirus. This is shown in an interim report of the research into the impact of the virus, carried out by RIVM and Nivel, the Dutch institute for health research.

Source: https://www.ad.nl/binnenland/onderzoek-wijst-uit-dat-nederlanders-vertrouwen-hebben-in-aanpak-van-het-coronavirus~a29de95e/ (Photo adjusted)

The Dutch are satisfied with the government's corona approach

Picture of Hugo de Jonge (Minister of Health, Welfare and Sport) and Prime Minister Rutte

The majority of the Dutch agree with the way in which the government is tackling the corona crisis. That is the result of a study by research agency Kantar. Last week, confidence in the government had even risen slightly compared to the week before.

Source: https://nieuws.nl/algemeen/20200331/nederlanders-tevreden-met-corona-aanpak-overheid/

The current study

The current study also shows that trust in the responsible authorities and experts has increased. This could mean that the events and changes in policy of the past two weeks have a positive effect on people’s trust in the authorities, healthcare and science.

Explanation

The increase in trust is due to the fact that **the responsible authorities and experts share the same views on how to manage the pandemic** and agree on key points. It is a fact that the rules can be complex, but the basis for **the policy is reliable** scientific research and the **communication** from the government **about the policy is clear**.

To know if you have seen the information above, we ask you a question about this:
According to the information above, has the level of trust in the responsible authorities and experts increased, decreased or remained the same?

1. Increased
2. Decreased
3. Remained the same

1.3.1.2 Social Norm

Social Norm low condition

Before we continue to the third survey, we would like to share a couple of news articles with you.

Busy on Ascension Day, parks vacated

Picture of an enforcer in a busy park

In several regions in the Netherlands, the beautiful weather on Ascension Day led to crowds. Roads and areas are closed to prevent the influx of day trippers.

Source: https://nos.nl/artikel/2334600-drukte-op-hemelvaartsdag-parken-ontruimd.html

Poll: support for measures is declining among the Dutch

Picture of busy beach (photo adjusted)

The Dutch are less concerned about the health risks of the corona pandemic. As a result, support for social distancing and the intelligent lockdown is decreasing, according to the Corona Impact Monitor from research agency Motivaction.

Source: https://www.volkskrant.nl/nieuws-achtergrond/peiling-draagvlak-voor-maatregelen-neemt-af-onder-nederlanders~bd7f3285/ (Photo adjusted)

The current study

The current study also shows that people adhere less to the corona measures. The extent to which people find it important that others adhere to the corona measures has also decreased.

Explanation

The fact that people adhere less to the corona measures is because **an increasing amount of people believes that the measures can be loosened,** since the Intensive Care Unit statistics leave plenty of room for this and the economic damage will otherwise be irreparable.

To know if you have seen the information above, we ask you a question about this:
According to the information above, has the level of compliance with the corona measures increased, decreased or remained the same?

1. Increased

2. Decreased
3. Remained the same

Social Norm high condition

Before we continue to the third survey, we would like to share a couple of news articles with you.

99 percent of the Dutch say they keep a distance of 1.5 meters

Picture of sign with the text ‘keep distance’

Almost all Dutch people adhere to the behavioral rules that must prevent infection with the corona-virus. 99 percent says they keep the requested 1.5 meter distance from others, 97 percent wash their hands more often and 93 percent stays at home as much as possible. More than half addresses others about violating these rules.

Source: https://www.nporadio1.nl/onderzoek/22800-99-procent-van-de-nederlanders-zegt-1-5-meter-afstand-te-houden

The Dutch stay at home despite beautiful spring weather

Picture of two people walking outside in nature

Despite the beautiful spring weather, the Netherlands seems to be following the advice to stay at home as much as possible. According to various regions and authorities, it has been quiet today in the nature reserves.

Source: https://www.rtlnieuws.nl/nieuws/nederland/artikel/5081001/rust-recreatiegebieden-oproep-thuisblijven-corona-verspreiding

The current study

Despite the fact that some places are getting crowded, research shows that the vast majority of people still comply with the rules. The current study also shows that people increasingly adhere to the corona measures. The extent to which people find it important that others adhere to the corona measures has also increased.

Explanation

The fact that people adhere more to the corona measures is because **an increasing amount of people believes that the measures are proportional to the current phase of the corona crisis**. Slightly looser where possible, but careful, to avoid later regrets, as Prime Minister Rutte says.

To know if you have seen the information above, we ask you a question about this: According to the information above, has the level of compliance with the corona measures increased, decreased or remained the same?

1. Increased
2. Decreased
3. Remained the same

1.3.1.3 T3 control condition

Before we continue to the third survey, we would like to share a couple of news articles with you.

Record number of new Netflix subscribers due to coronavirus

Picture of Netflix tv screen and smart phone with Netflix on its screen

Netflix has never welcomed as many new subscribers in a quarter as in the first three months of 2020: 15.8 million worldwide, the streaming service announced on Wednesday. The record increase is the result of the corona measures, which means that many people have to stay at home.

Source: https://www.nu.nl/tech/6046377/recordaantal-nieuwe-abonnees-voor-netflix-door-coronavirus.html

To know if you have seen the information above, we ask you a question about this: According to the information above, how many new subscribers has Netflix welcomed in the first quarter of 2020?

1. 2,3 million
2. 15,8 million

3. 33,4 million

1.3.2 Interventions T5

1.3.2.1 Measures Support

Measures Support low condition

Before moving on to the final questionnaire, we would like to share some news items with you.

'Corona measures actually cause more deaths in the long term'

Picture of Ira Helsloot (professor Governance of Safety and Security at the Radboud University)

The measures taken to combat the coronavirus cost more lives than the deaths they prevent. "Only you don't see the deaths that will fall in two years."

Source: https://www.ad.nl/binnenland/coronamaatregelen-veroorzaken-op-langere-termijn-juist-meer-doden~a0f0aa10/

Confusion about corona rules in shops are disastrous for support for corona measures

Picture of list with corona rules on door of clothing shop

While in the one shop a shopping basket is mandatory, in another it requires a cart. Disinfect hands here, customer card there. Many retailers mean well, but the tangle of shop rules is disastrous for the support of the corona measures. "People have to understand the bigger picture if you want them to keep understanding."

Source: https://www.ad.nl/binnenland/wirwar-aan-winkelregels-funest-voor-draagvlak-coronamaatregelen~a141c30c/
(Photo adjusted)

The current study

The current study also shows that the **corona measures** are seen as **exaggerated and unnecessary**. **Support** for current measures remains **low**.

Explanation

The decreasing support for the corona measures is due to it becoming increasingly clear what the **negative effects of the measures** are. People also find the measures **unclear and too**

strict for the phase of the corona crisis we are currently in. Many people feel that after the sacrifices in the early stages of the pandemic, it is **time for more** individual **freedom**.

To know if you have seen the information above, we ask you a question about this: According to the information above, are the effects of corona measures positive, negative or neutral?

1. Positive
2. Negative
3. Neutral

Measures Support high condition

Before moving on to the final questionnaire, we would like to share some news items with you.

Lockdowns save the lives of 'millions of Europeans'

Picture of Eiffel tower in Paris
and Big Ben in London

The strict lockdowns introduced in several European countries have saved millions of lives. British research shows that the number of deaths in Europe in early May, without lockdowns, would have risen to 3.1 million. In reality, 135,000 people had died at the time.

Source: https://www.rtlnieuws.nl/nieuws/buitenland/artikel/5147006/lockdown-studie-levens-3-miljoen-mensen-europa-gered

RIVM: 90 percent of ICU admissions prevented by corona measures

Picture of health care professional
at intensive care unit (photo adjusted)

If measures had not been taken to contain the spread of the new coronavirus, an estimated 23,000 critically ill patients would have required ICU care. That is what RIVM director Jaap van Dissel told the House of Representatives this afternoon.

Source: https://www.ad.nl/politiek/br-rivm-90-procent-ic-opnames-voorkomen-door-coronamaatregelen~a2dd5dc5/ (photo adjusted)

The current study

The current study also shows that the **corona measures** are seen as **effective and necessary**. **Support** for current measures remains **high**.

Explanation

The decreasing support for the corona measures is due to it becoming increasingly clear what the **positive effects of the measures** are. As a result of the measures we have seen a sharp decrease in mortality rates and ICU admissions. People also find the current measures are a

good fit with the phase of the corona crisis we are currently in. We have **avoided even worse consequences together** and now it is a matter of **persistence**.

To know if you have seen the information above, we ask you a question about this:
According to the information above, are the effects of corona measures positive, negative or neutral?

1. Positive
2. Negative
3. Neutral

1.3.2.2 Economic Consequences

Economic Consequences low condition

Before moving on to the final questionnaire, we would like to share some news items with you.

Economic impact of the coronavirus may be less serious than expected

Picture of empty terrace from the catering industry (photo adjusted)

The economic impact of the new coronavirus may not be as serious as the rating agency S&P Global recently predicted. The International Monetary Fund (IMF) is a bit more cautious when it comes to estimating the damage, although Kristalina Georgieva (IMF) emphasized this weekend that the damage will be more serious if the virus continues to spread.

Source: https://www.telegraaf.nl/financieel/1582437355/economische-impact-coronavirus-valt-mogelijk-mee (photo adjusted)

ABN AMRO: corona recession becomes deep, but short

Picture of travelers at
an airport (photo adjusted)

The recession caused by the corona pandemic will be deep, but short-lived. That is what the economists of ABN Amro estimate. They assume that Dutch GDP will shrink by 3.5 percent this year.

Source: https://www.rtlnieuws.nl/economie/artikel/5072101/recessie-corona-covid19-abn-amro-economie-daalt-krimp-scenario-crisis (photo adjusted)

The current study

The current study also shows that people expect the economic damage to be **less serious than expected**. The pandemic will have adverse effects on our economy, but these will be **temporary**. People are **optimistic** about the economic impact of the pandemic on them personally. The economic consequences for family and friends are also estimated to be relatively small.

Explanation

The economic consequences of the Dutch policy during the corona pandemic are now **estimated to be smaller** than previously expected. The **government's financial support** is

more than **sufficient** to deal with the consequences of the pandemic. The **economy will** also **quickly recover** due to the recent loosening of measures.

To find out whether you have seen the information above, we ask you a question about this: According to the information above, are the economic consequences more serious than expected, less serious than expected or neutral?

1. More serious than expected
2. Less serious than expected
3. Neutral

Economic Consequences high condition

Before moving on to the final questionnaire, we would like to share some news items with you.

CPB: unprecedented economic shrinkage of 6 percent, unemployment doubles

Picture of people in shopping street

The Dutch economy is shrinking by 6 percent this year due to the corona outbreak. Next year there will be limited recovery with a growth of 3 percent. Unemployment will rise and will double in 2021, the Central Planning Bureau (CPB) expects. The CPB speaks of an unprecedented shrinkage and warns of great uncertainty about the course of the corona crisis.

Source: https://nos.nl/artikel/2337409-cpb-ongekende-krimp-van-6-procent-werkloosheid-verdubbelt.html

Economic blows due to coronavirus: 'We have not had everything yet'.

Picture of feet standing on floor sign with the text ‘keep distance’

"You can certainly say that this is the biggest economic crisis since the Second World War," says Peter Hein van Mulligen, chief economist at Statistics Netherlands. This morning Statistics Netherlands presented the results of the Dutch economy in the first quarter. And they did not look good, Van Mulligen sees. "I can't make it more fun." The first quarter of 2020 shows a shrinkage of 1.7 percent. After good results in January and February, a dramatic downturn in the last two weeks of March wiped out that prosperity.

Source: https://nos.nl/artikel/2334033-economische-klappen-door-coronavirus-we-hebben-nog-lang-niet-alles-gehad.html

The current study

The current study also shows that people expect that the **economic damage will be enormous**. The **economic consequences** of the pandemic, both personally and for family and friends, are estimated to be **very serious**.

Explanation

The **seriousness of the economic consequences** of the Dutch policy during the corona pandemic is becoming **increasingly clear**. Our **economy is shrinking** and **unemployment is rising**. There is even talk of the worst economic crisis since World War II.

To know whether you have seen the information above, we ask you a question about this: According to the information above, are the economic consequences very serious, less serious than expected or neutral?

1. Very serious
2. Less serious than expected
3. Neutral

1.3.2.3 T5 control condition

Before moving on to the final questionnaire, we would like to share some news items with you.

More car ownership or car use due to the corona crisis?

Picture of hands on steering wheel and phone in car

Due to the corona crisis, fewer people are using public transport and the sale of second-hand cars and motorcycles is on the rise. According to Karen van den Boom, CEO of SIXT Netherlands, people have started to think differently about the role of transport in our daily lives. NU.nl spoke with Van den Boom about how mobility providers respond to this.

Source: https://www.nu.nl/auto/6056501/door-coronacrisis-meer-autobezit-of-autogebruik.html

The **sale of used cars increased** as a result of the corona crisis.

Due to the corona crisis, fewer people are using public transport and the sale of second-hand cars and motorcycles is increasing. The corona crisis has an impact on how people think about the role of transport in our daily lives.

To know if you have seen the information above, we ask you a question about this: According to the information above, has the sale of used cars increased, decreased or remained the same as a result of the corona crisis?

1. Increased
2. Decreased
3. Remained the same

2. Additional information on analyses

2.1 Dropped out participants longitudinal study

Note that this section is identical to Chambon, Dalege [17]. Demographic information was compared between the longitudinal sample and the participants that dropped out of the study before the final wave (total drop-out n = 3,694). A chi square test of independence indicated that the proportion of participants that dropped out of the study differed by gender, χ^2 (1, N = 6,093) = 6.55, p = .011, and illness, χ^2 (1, N = 6,093) = 34.53, $p < .001$. Specifically, men and participants suffering from a condition that could worsen the consequences of COVID-19 were more likely to participate in the current study up and until the final wave. No significant differences between the longitudinal sample and drop-outs were found for education and smoking. A Mann-Whitney U test indicated that the age was significantly higher in the longitudinal sample (*Mdn* = 56) than in the group of participants that dropped out of the study (*Mdn* = 47), U = 5,529,364.50, $p < .001$, which implies that the average age of the longitudinal sample is higher than the average Dutch population.

The Network Comparison Test [18] was conducted to formally test for differences in network structure at the first measurement between respondents from the longitudinal sample and respondents that dropped out of the study after the first measurement. Results revealed network invariance (p <.001), indicating that not all edges are equal between the networks of the first measurement of the longitudinal sample and respondents that dropped out. Further investigation into specific edge differences reveals that, after applying a Holm correction for multiple testing, only two edges differ significantly at the first measurement between the longitudinal and dropout sample. More specifically, the edges that differ significantly are somatic symptoms and self-exempting beliefs (i.e., weak positive edge is stronger in dropout sample), and between somatic symptoms and social norm (i.e., weak negative edge is absent in dropout sample). Finally, results indicated a significant difference in global strength (i.e.,

overall connectivity of the networks) between the longitudinal sample and respondents that dropped out of the study (global strength is 2.61, p = .036).

2.2 Node construction and Principal Axis Factoring

Note that this section is identical to the section on node construction in Chambon, Dalege [17], with addition of variables that were measured once (i.e., Consideration of Future Consequences, Resilience and Coping).

The analysis for node construction was conducted with the largest and most diverse sample available: wave 1, including all participants that completed the first sample (i.e., no drop-out). The survey items were combined to form nodes: the combination of items was either predetermined by validated scales or a fixed operationalization or identified through Principal Axis Factoring (PAF; see Table S2). PAF was conducted with Oblimin rotation given the expected intercorrelation between items. Extraction of components was based on eigenvalues greater than one. The PAF results are discussed below. Items presented in italic were excluded from the node.

Table S2. Overview of nodes and the approach to the selection of items per node

Nodes	Approach to combining items in node
**Compliance (T1)**	Predetermined - Operationalized with the recommended behavioral measures, PAF confirmed single component
**Risk Perception**	Predetermined by product of likelihood and severity
**Health Risk**	Predetermined – Two item construct
**Economic Consequences**	Predetermined – Two item construct
**Self-exempting Beliefs**	PAF – Single component
**Negative Affect**	PAF – Component identified in items on affect
**Compassion**	PAF – Component identified in items on affect
**Worries Virus**	PAF – Component identified in items on worries
**Worries Measures**	PAF – Component identified in items on worries
**Vaccination Intention**	Predetermined single item node
**Measures Support**	PAF – Component identified in items on attitudes toward corona measures
**Measures Ease**	PAF – Component identified in items on attitudes toward corona measures
**Social Norm**	Predetermined – Two item construct
**Control Infection**	Predetermined – Two item construct
**Self-efficacy**	Predetermined single item node
**Involvement**	PAF – Single component
**Perceived Knowledge**	Predetermined single item node
**Trust**	PAF – Single component
**Consideration of Future Consequences**	Predetermined - Validated scale
**Resilience**	Predetermined - Validated scale
**Coping**	Predetermined - Validated scale
**Health General**	Predetermined single item node
**Health change Physical**	Predetermined single item node
**Health change Mental**	Predetermined single item node
**Healthy Lifestyle**	Predetermined - Operationalized with basic items on diet, exercise and sleep, PAF confirmed single component
**Mental Wellbeing**	Predetermined - Validated scale
**Loneliness**	Predetermined - Validated scale
**Somatic Complaints**	Predetermined - Validated scale
**Depressive Complaints**	Predetermined - Validated scale
**Anxiety Complaints**	Predetermined - Validated scale

Compliance

Compliance was predetermined through operationalization with the recommended behavioral measures. Kaiser-Meyer-Olkin Measure of Sampling Adequacy was .78 and Bartlett's test of sphericity was significant, $\chi^2(df = 10) = 8{,}429.90$, $p < .001$, supporting a rationale for performing PAF with the five items for *Compliance* in the first wave (i.e. a general item and four specific behaviors). The results suggested a single component for the items on compliance, resulting in the node *Compliance* with the four items shown below.

No.	Survey items	Compliance Component load
1	I comply with the corona measures.	.837
2	Stay at home as much as possible.	.613
3	Keep 1.5 meters away from others.	.735
4	Wash your hands regularly with soap and water.	.545
5	Cough and sneeze into the inside of your elbow.	.444

Self-exempting Beliefs

Kaiser-Meyer-Olkin Measure of Sampling Adequacy was .54 and Bartlett's test of sphericity was significant, $\chi^2(\text{df} = 3) = 1{,}320.13$, $p < .001$, not convincingly supporting a rationale for performing PAF with the three items for *Self-exempting Beliefs*. Nevertheless, the results suggested excluding the item 'I think infections are good for building up group immunity (protection)', because of its low Component load. This is supported by an increase in scale reliability after deletion (three items $a = .47$, if item deleted $a = .57$; resulting in $r_{sb} = .57$). As a result, the node *Self-exempting Beliefs* consisted of item 1 and 2 (see below).

No.	Survey items	Self-exempting Beliefs Component load
1	I will not get infected with the coronavirus because I never get the seasonal flu (influenza) either.	.505
2	I think I am already immune (protected) against the coronavirus.	.780
3	*I think infections are good for building up herd immunity (protection).*	*.242*

Negative Affect and Compassion

Kaiser-Meyer-Olkin Measure of Sampling Adequacy was .90 and Bartlett's test of sphericity was significant, $\chi^2(\text{df} = 36) = 24{,}219.84$, $p < .001$, supporting a rationale for performing PAF with the nine items covering affect. Compassion was identified as a separate component. This resulted in two nodes: *Negative Affect*, consisting of the items 1 to 8 shown below, and the single-item (no. 9) node *Compassion.*

No.	Survey items	Negative Affect Component load	Compassion Component load
1	The corona pandemic is making me angry.	.729	
2	The corona pandemic is making me sad.	.475	
3	The corona pandemic is making me feel confused.	.678	
4	The corona pandemic is making me feel uncertain.	.649	
5	The corona pandemic is making me feel overwhelmed.	.472	
6	The corona pandemic is making me feel frustrated.	.890	
7	The corona pandemic is making me fearful.	.572	
8	The corona pandemic is making me feel out of control.	.628	
9	The corona pandemic is making me feel compassion.		.511

Worries Virus and Worries Measures

Kaiser-Meyer-Olkin Measure of Sampling Adequacy was .84 and Bartlett's test of sphericity was significant, $\chi^2(df = 55) = 13{,}097.02$, $p < .001$, supporting a rationale for performing PAF with 11 items covering worries. The results indicated two components for items on worries: items on worries about the virus and its effects (*Worries Virus*) and items on worries about the effects of the measures taken to manage the pandemic (*Worries Measures*). The item 'I worry about society becoming increasingly egoistic' was excluded because this item had a low Component load on both components. This resulted in two nodes: the node *Worries Virus* that consisted of item 1 to 4 shown below, and the node *Worries Measures* that consisted of items 5 to 10.

No.	Survey items	Worries Virus Component load	Worries Measures Component load
1	I worry about getting infected with the coronavirus.	.718	
2	I worry about infecting others with the coronavirus.	.511	
3	I worry about losing someone I love.	.695	
4	I worry about the health care system overloading.	.627	
5	I worry about what staying at home a lot will do to my health.		.716
6	I worry about the schools closing.		.326
7	I worry about a recession.		.301
8	I worry about limited access to food.		.384
9	I worry about losing my job.		.427
10	I worry about getting lonely.		.633
11	*I worry about society becoming increasingly egoistic.*	*.269*	*.218*

Measures Support and Measures Ease

Kaiser-Meyer-Olkin value was .90 and Bartlett's test of sphericity was significant, χ^2(df = 45) = 31,141.32, $p < .001$, supporting a rationale for performing PAF with the 10 items on attitudes toward the corona measures. The item 'I think the corona measures will prevent the spread of the coronavirus' initially loaded on a third component, but due to the low eigenvalue for this component (1.003) we reran the PAF with two fixed components, resulting in this item loading on the component *Measures Support*. Furthermore, Cronbach's alpha of the node *Measures Support* did not require the exclusion this item (*a* = .90, if item deleted *a* = .91). The item '*I find the corona measures... Insufficient - Overdone'* was excluded, because this item has a low component load on the *Measures Support* component. This resulted in two nodes: the node *Measures Support,* consisting of the items 1 to 7 shown below, and the node *Measures Ease,* consisting of the items 8 and 9.

**No.**	**Survey items**	**Measures Support Component load**	**Measures Ease Component load**
1	I find the corona measures... Senseless – Sensible.	.869	
2	I find the corona measures... Useless – Useful.	.877	
3	I find the corona measures... Unnecessary – Necessary.	.901	
4	I find the corona measures... Unfair – Fair.	.647	
5	I find the corona measures... Unacceptable – Acceptable.	.776	
6	I think the corona measures will prevent the spread of the coronavirus.	.497	
7	As a society, we must do whatever is necessary to prevent the spread of the coronavirus.	.654	
8	I find the corona measures... Unpleasant – Pleasant.		.661
9	I find the corona measures... Difficult – Easy.		.774
10	*I find the corona measures... Insufficient – Overdone.*	-*.242*	

Involvement

Kaiser-Meyer-Olkin value was .73 and Bartlett's test of sphericity was significant, χ^2(df = 6) = 7,774.14, $p < .001$, supporting a rationale for performing PAF with the four items

on the degree to which participants are involved in the corona pandemic. The items loaded on one component, resulting in the node *Involvement* with the items 1 to 3 shown below. The item '*To what extent do you feel you can avoid the news about the corona pandemic?'* was excluded, because this item has a low component load on the *Involvement* component.

**No.**	**Survey items**	**Involvement Component load**
1	How important is the topic of corona pandemic to you?	.832
2	To what extent does the news about the corona pandemic have your attention?	.807
3	How much do you think about the corona pandemic?	.754
4	*To what extent do you feel you can avoid the news about the corona pandemic?*	*-.176*

Trust

Kaiser-Meyer-Olkin value was .73 and Bartlett's test of sphericity was significant, χ^2(df = 6) = 14,759.76, $p < .001$, supporting a rationale for performing PAF with the four items on trust in actor relevant to manage the corona pandemic. The results suggested a single component for the items on trust, resulting in the node *Trust* with the four items shown below.

**No.**	**Survey items**	**Trust Component load**
1	I trust the authorities to adequately manage the corona pandemic.	.883
2	I trust RIVM during the corona pandemic.	.901
3	I trust health care professionals during the corona pandemic.	.543
4	I trust science during the corona pandemic.	.767

Healthy Lifestyle

Healthy Lifestyle was predetermined through operationalization with basic items on diet, exercise and sleep.[1] Kaiser-Meyer-Olkin value was .60 and Bartlett's test of sphericity was significant, χ^2(df = 3) = 1,171.04, $p < .001$, not convincingly supporting a rationale for

[1] The survey also contained items on smoking and alcohol consumption, but these items were excluded from further analysis due to the large amount of missing values. These items loaded on another component than *Healthy Lifestyle*.

performing PAF with the three items on healthy lifestyle change compared to before the corona pandemic. Nevertheless, in accordance with the predetermined operationalization the results suggest a single component for the items. This is confirmed by the result that scale reliability statistics (*a* = .49) decrease when excluding items. This resulted in the node *Healthy Lifestyle* consisting of the three items shown below.

**No.**	**Survey items**	**Healthy Lifestyle Component load**
1	I've been eating in the past two weeks, compared to before the corona pandemic... Less healthy – healthier.	.607
2	I've been exercising in the past two weeks, compared to before the corona pandemic… Less – More.	.521
3	I've been sleeping for the past two weeks, compared to before the corona pandemic... Worse – Better.	.404

2.3 Detailed description per node

This section provides detailed description of each node in the COVID-19 broad attitude network, its interpretation and the scale reliability as observed in the current study. Note that this section is similar to the node description in Chambon, Dalege [17], with addition of variables on individual differences that were measured once (i.e., Consideration of Future Consequences, Resilience and Coping).

Compliance

Compliance with behavioral measures was operationalized through the preventive behaviors as recommended by the Dutch national government [19] and National Institute for Public Health and the Environment [20]. We measured the (self-reported) extent to which participants adopted the preventive behaviors that were advised to the general public, regardless of symptoms (i.e., hygiene behaviors and physical distancing). Due to changes in

the recommended behaviors and policy, the items underlying this node also changed over time. Specifically, the node consisted of five items in wave 1 (a = .75), of which one item was adapted in wave 2, and then a sixth item was added in wave 3 (resulting in six items from wave 3 onwards). Higher scores indicate that participants reported to display those behaviors much more throughout time and therefore reflect a higher degree of compliance with the behavioral measures.

Attitudes

The following nodes consisted of items that measured the different elements of attitudes relevant for the pandemic and the behavioral measures.

Based on prior research into psychological factors during the COVID-19 pandemic [21], we included items that formed the cognitive nodes *Risk Perception*, *Health Risk* (r_{sb} = .59) and *Economic Consequences* (r_{sb} = .75). The node *Risk Perception* (i.e., likelihood and severity of infection for oneself) was calculated by multiplying the scores on the two items [22]. Higher scores on the nodes *Risk Perception* and *Health Risk* indicate a higher perceived risk of infection and severe health consequences due to an infection, respectively. Higher scores on *Economic Consequences* indicate that participants expected the economic consequences of the pandemic to be more severe. Additionally, the items on *Self-exempting Beliefs* (r_{sb} = .57) resulted in a node for which a higher score reflects stronger convictions about not being susceptible to the coronavirus.

The items on affect resulted in two nodes: *Negative Affect* (e.g., anger, anxiety and confusion; a = .89) and *Compassion* (single item). Higher scores indicate that the pandemic caused these emotions to be experienced more often. The construct worries resulted in the nodes *Worries Virus* (a = .73), with items encompassing worries about events during the pandemic that resulted directly from the virus (e.g., getting infected, losing someone they

love), and *Worries Measures* (*a* = .67), with items concerning worries about events that resulted from the measures taken due to the virus (e.g., overloading the health care system, a recession). Higher scores reflect more worries.

Three behavioral attitude nodes were identified. The first node was formed by a single item that measured the intention to get vaccinated if a vaccine becomes available (*Vaccination Intention),* with a higher score indicating more intention. The items on attitudes toward the behavioral measures (general items and semantic differential scale items) resulted in two nodes: *Measures Support* (*a* = .90) and *Measures Ease* (r_{sb} = .68), with items measuring the participants' support for the behavioral measures aimed at preventing the spread of the coronavirus, and the extent to which these measures were perceived as easy to comply to, respectively. Participants with a higher score on these nodes reported more support for and perceived ease of the behavioral measures.

Additional psychological factors

The following nodes were included in addition to attitudes.

The node *Social Norm* (r_{sb} = .76) consisted of items measuring the prescriptive and descriptive social norm, combinedly forming one node for which a higher score implies participants perceived a stronger norm towards compliance with the behavioral measures.

Analysis of the items relating to perceived control resulted in two nodes: *Control Infection* (r_{sb} = .61), with items measuring the extent to which people felt they could avoid an infection with the coronavirus, and *Self-efficacy*, a single item measuring whether people know how to protect themselves from the virus. Higher scores indicate more perceived control over an infection with the coronavirus and perceived self-efficacy, respectively.

The node *Involvement* (*a* = .84) consisted of items for which a higher score means participants perceived themselves to be more actively involved in the corona pandemic (e.g.,

allocated importance and watching the news), which is perceived as a dimension of attitude strength.

The node *Perceived Knowledge* was formed by a single item with higher scores meaning participants reported more (self-perceived) knowledge about the pandemic.

Trust (*a* = .86) was measured with items that formed a node by combining general trust in four actors that are crucial in the corona pandemic (adapted to the Netherlands): the authorities, the Dutch National Institute for Public Health and the Environment (RIVM), health care professionals and science. Higher scores reflect more trust in these actors.

Individual differences were included by adopting three available scales with items that measured personality aspects that were expected to be a relatively stable indication of people's response to the current pandemic, and therefore included only at wave 1. More specifically, we included the following scales as nodes: *Consideration of Future Consequences* [subset of five items, a = .34, adopted from 23][2], *Resilience* [a = .84; adopted from 24] and *Coping* [subset of 10 items, a = .83, adopted from 25, based on Kalisch et al., 2020], with a higher score indicating more consideration of future aspects, resilience and positive coping, respectively.

Health and well-being nodes

Finally, several health- and well-being-related nodes were included into the broader network with attitudes and additional psychological factors. More specifically, we incorporated health-related nodes that could influence people's vulnerability (e.g., general health, illness) and thus fear of this pandemic, and thereby possibly affecting compliance.

[2] Although the scale reliability of some nodes was lower than one would normally prefer, these were interpreted as sufficient given our objective of measuring evaluations instead of designing reliable measurement scales (Dalege J, Borsboom D, van Harreveld F, van den Berg H, Conner M, van der Maas HLJ. Toward a formalized account of attitudes: The Causal Attitude Network (CAN) model. Psychological Review. 2016;123(1):2–22.). Reasons for the low scale reliability found in this study for the (subset of) items measuring *Consideration of Future Consequences* are not clear.

Furthermore, we included health-related nodes that could be influenced by the pandemic and associated behavioral measures (e.g., healthy lifestyle, psychological complaints).

Health was measured with the single item node *General Health,* consisting of an overall score participants assign to their health, with a higher score indicating better health. The nodes *Health change Physical* and *Health change Mental* indicate how participants rated their physical and mental health at the moment of completing the survey compared to before the pandemic. Higher scores indicate participants experienced an improvement, whereas lower scores indicate they reported a decline in physical and mental health. The node *Healthy Lifestyle* (a = .49) consisted of items measuring change in lifestyle (i.e., diet, exercise and sleep), with higher scores indicating improvement and lower scores indicating deterioration in healthy lifestyle. Furthermore, participants were asked to indicate whether they smoke (node *Smoking)* and whether they suffer from a health condition known to increase the health risks of COVID-19 (node *Illness)*.

The (S)WEMWBS [Short Warwick Edinburgh Mental Well-being Scale; 27] was adopted to form the node *Mental Well-being* (a = .84). This node was calculated by the sum of the items, with a higher score indicating higher well-being. The node *Loneliness* (a = .76) was measured with a shortened version of the (emotional and social) loneliness scale from de Jong Gierveld and van Tilburg [28]. We adjusted the answer scale and specified a timeframe of two weeks to meet the objectives of the current study. Higher scores indicate more loneliness.

Psychological complaints were measured with the Brief Symptom Inventory 18 [BSI-18; 29]. This scale measures three important areas of psychological distress with 18 items: somatic, depressive and anxiety symptoms. It should be noted that the answer scale of the BSI-18 was altered, and a timeframe of two weeks was specified in the items. This might invalidate the clinical norm score of the BSI-18, therefore from here on we will use the term

psychological complaints instead of symptoms. This resulted in three nodes (i.e., *Somatic Complaints* [*a* = .84], *Depressive Complaints* [*a* = .90] and *Anxiety Complaints* [*a* = .92]), for which higher scores indicate more complaints.

2.4 R script

See OSF (https://osf.io/bhuvw/) for the .R file containing the R script. The R script [30] was based on the tutorial papers of Dalege, Borsboom [31] and Haslbeck and Waldorp [32].

2.5 Additional information on Network Analysis

The communities in the networks were determined through a community stability and detection analysis with the cluster walktrap algorithm. Stability analysis was conducted by repeating the community analysis (1000 iterations) and calculating how often different nodes belonged to the same community. This resulted in a score between 0 and 1 for each combination of nodes, in which 0 meant that these nodes never belonged to the same community, and 1 that these nodes belonged to the same community in every iteration. Subsequently, a community detection analysis was conducted by identifying the communities with nodes that belonged to that community in over 90 percent of the iterations. The output of the community analyses is provided in section 3.1.2..

We used the package *qgraph* [33] to visualize the graphs, *bootnet* [34] for the stability and accuracy measures and *igraph* [35] for the community detection.

3. Additional information on results

3.1 Part 1 - Network structure

3.1.1 Detailed descriptive account of COVID-19 broad attitude network

The COVID-19 broad attitude network, obtained through nodewise regression, is shown in Fig S1a (left). Nodes represent the measured psychological factors. In general terms, the right section of the network displays the (psychological) health nodes. The left half of the network is comprised of the cognitive and behavioral attitude nodes and the additional psychological nodes (e.g., social norm, perceived knowledge, trust and perceived control).

The colored groups represent communities (i.e., clusters with higher interconnectedness) that consist of nodes that were more connected to each other than to other nodes. The yellow cluster indicates that attitudes, the additional social psychological nodes and compliance with behavioral measures form a community in the COVID-19 broad attitude network as they were highly interconnected. This also applies to the orange community with nodes on mental health and well-being and the purple community with nodes on current and perceived risk of physical health.

The edges represent linear relations between two nodes after controlling for every other node in the network. These edges are associations, meaning that the direction of the relation is not determined. Interpretation guidelines can be found in the caption of Fig S1. Edge weights are regression coefficients that represent the strength of a relation between two nodes after removing effects from all other nodes in the network. Edges with weights below the value of .10 are omitted from the figure to facilitate readability (see section 3.1.3 for network without threshold). The edges discussed in this section had sufficient estimation accuracy and their edge weights are reported in parentheses. A complete overview of the edge weights and their accuracy is provided in this supplement (3.1.4, 3.1.6). In order to

provide a brief descriptive account of the COVID-19, we focus on the behavioral (i.e., compliance) and health elements.

In total, 24 of the 35 nodes had a direct relation with *Compliance*. The nodes in the COVID-19 broad attitude network that showed the strongest relation with *Compliance* were *Gender* (.28), *Measures Support* (.24) and *Self-efficacy* (.13). This indicates that compliance was positively associated with being female, support for the behavioral measures and perceived self-efficacy. The edges between *Compliance* and *Gender,* and *Compliance* and *Measures Support,* were of comparable weight, as indicated by edge weight and formally tested with an edge difference test (see 3.1.6). Furthermore, these edges were both significantly stronger than the edge between *Compliance* and *Self-efficacy.* Regarding the nodes on individual differences, *Compliance* was positively related to *Consideration of Future Consequences* and *Coping* (both .07).

Besides direct relations between nodes, as shown in the network figure and the edge weight table in this supplement, nodes can be indirectly related (i.e., distance of two edges). *Compliance* in the COVID-19 broad attitude network was indirectly related to the nodes that were directly related to *Gender, Measures Support* and *Self-efficacy*. Nodes that were indirectly related to *Compliance* through a positive relation with *Measures Support* were *Trust* (.44; significantly strongest positive edge[3]) and *Measures Ease* (.25; significantly second strongest positive edge), but also *Worries Virus* (.16), *Vaccination Intention* (.15) and *Involvement* (.11). These nodes were also directly related to *Compliance* with an edge weight below .10 and therefore omitted from the network figure. This indicates that compliance with the behavioral measures during the COVID-19 pandemic was both directly and indirectly

[3] This indicates that the relation between *Measures Support* and *Trust* was significantly stronger than the other relations that *Measures Support* has, which is of relevance for the indirect influence on the node of focus (in this section *Compliance*); the node of focus (here *Compliance*) was therefore excluded in the formal ranking of the relations. Nodes that do not contain an addition with information on the order of edge strength are of comparable strength.

Fig S1. Psychological broad COVID-19 attitude network. a) Network obtained through nodewise regression. The nodes represent the measured psychological factors and the edges represent the relations between nodes. For the binary nodes (marked with ^), a positive relationship indicates that increasing the other node results in a higher probability for category one of the binary node (i.e., Gender 1 = Female; Education 1 = Higher; Smoking 1 = Yes; Illness 1 = Yes). Positive edges (blue) represent excitatory influence and negative edges (red) represent inhibitory influence. The strength of the relation is indicated by the edge weight (visualized by edge width and color density). Edges with edge weights below the value of .10 are omitted to facilitate readability; b) The centrality measure 'Strength' for each node in the COVID-19 broad attitude network. This measure represents the direct effect of a specific node on the network and is calculated by the sum of the absolute edge weights of the relations a specific node has with connected nodes.

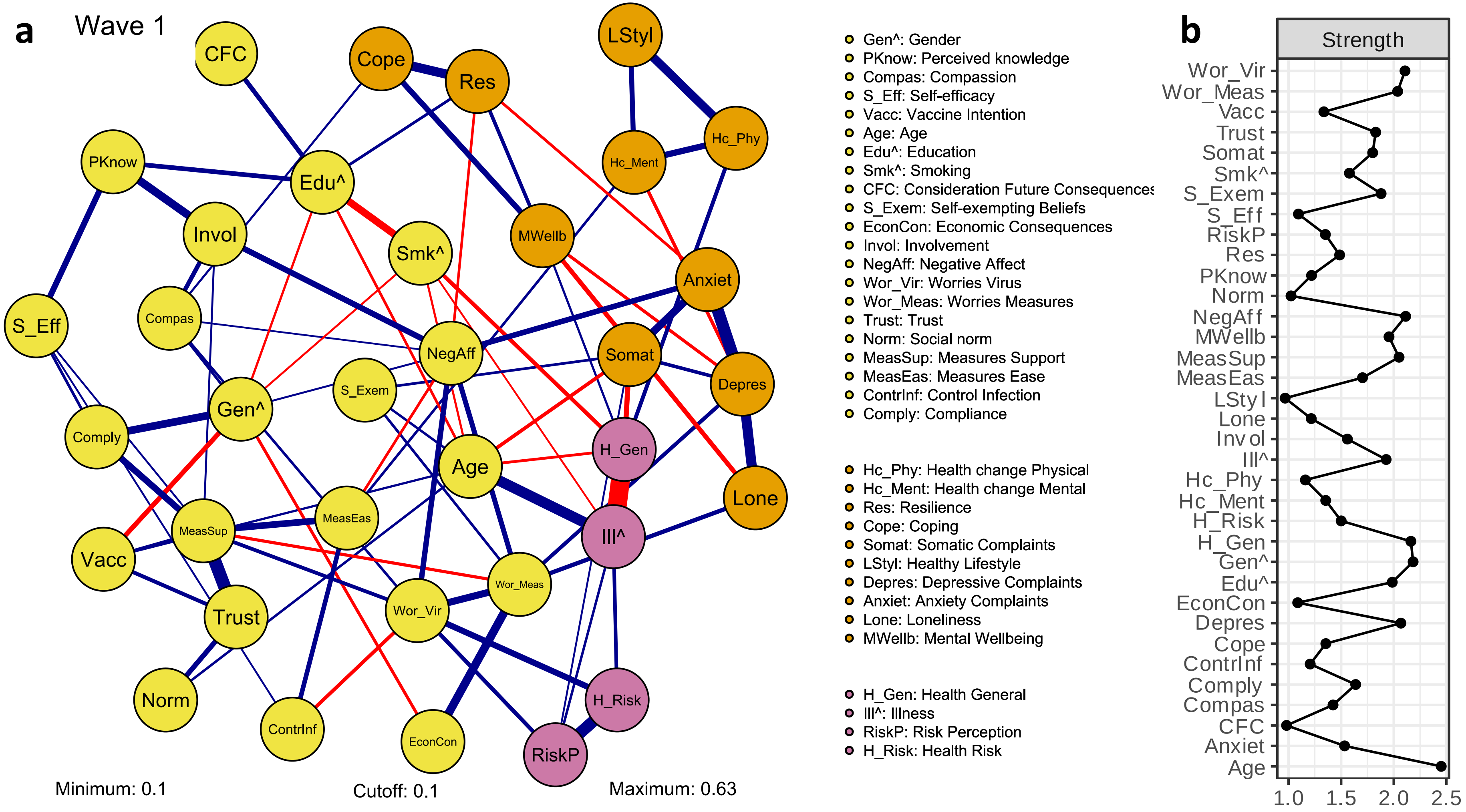

related to the degree to which participants trust the relevant authorities, perceive the measures as easy to comply with, worry about the consequences of the virus, intent to get vaccinated and are involved in the pandemic. *Compliance* was also directly and indirectly related to *Worries Measures* through a negative relation with *Measures Support* (-.14), indicating that compliance with the behavioral measures was indirectly related to how much participants worried about the consequences of the measures. Although causal inferences cannot be made, if such a causal relation were present, this could imply that an increase in worries about the measures can lead to a decrease in support for the measures and therefore a decrease in compliance with the measures.

Regarding well-being during the COVID-19 pandemic, a total of 28 of the 35 nodes had a direct relation with *Mental Well-being*. The relatively important nodes that were directly associated with *Mental Well-being* were *Coping* (.22; significantly strongest positive edge), *Resilience* (.16; significantly second strongest positive edge) and *Health General* (.12), but also *Loneliness* (-.19; significantly strongest negative edge) and *Depressive Complaints* (-.15). This implies that higher mental well-being during the COVID-19 pandemic was related to a positive coping strategy, more resilience, and better perceived general health, and with less experienced loneliness and depressive complaints. Two pairs of these nodes that were connected to *Mental Well-being* were also directly connected among each other, namely *Coping* and *Resilience* (.33), and *Depressive Complaints* and *Loneliness* (.36). Such a triangle is a particular motif that lends support to the idea that there is a reinforcing structure.

The COVID-19 broad attitude network also showed that *Vaccination Intention* was higher among men (-.20) and positively associated with *Trust* (.17) and *Age* (.12). Another salient detail was that the node *Negative Affect*, positioned at the center of the network, had eight relations to other nodes in the network: the significantly strongest, positive edges of comparable weight with *Involvement* (.23), *Anxiety Complaints* (.22), *Worries Virus* (.21) and

Worries Measures (.20), followed by the significantly weaker edges of comparable weight with *Gender* (.11) and *Compassion* (.10), and negative edges with *Measures Ease* (-.13) and *Resilience* (-.13).

Centrality

Fig S1b (right) presents the node strength measure for the psychological COVID-19 broad attitude network. It is calculated by the sum of the absolute edge weights of relations a specific node has with connected nodes and is related to the position of nodes in the network. The stability of this centrality measure for the COVID-19 broad attitude network was sufficient (see next section).

The relatively moderate strength of the node *Compliance* (1.64) in the network suggests that this node's conditional association with other nodes in the COVID-19 broad attitude network is moderate. Concerning strength of the nodes that had a direct relation with *Compliance*: the nodes *Gender* (2.18) and *Measures Support* (2.05) had the highest and comparable node strength, followed by the significantly lower node strength of *Self-efficacy* (1.09). This suggests relative high importance of *Gender* and *Measures Support* for the network due to the amount and weight of edges with other nodes.

The relatively high node strength of *Mental Well-being* (1.95) in the COVID-19 broad attitude network indicates relatively high conditional association with other nodes. Regarding the strength of the nodes that had a direct relation with *Mental Well-being* in the network, the nodes *Health General* (2.16) and *Depressive Complaints* (2.07) had the highest and comparable node strength, followed by the significantly lower but mutually comparable node strengths of *Resilience* (1.49) and *Coping* (1.35). Subsequently, the significantly lower node strength of *Loneliness* (1.22) follows, differing significantly from *Resilience,* but not from *Coping*. This indicates that, regarding nodes related to *Mental Well-being*, perceived general

health and depressive complaints have the relatively highest conditional association with other nodes in the network.

Interestingly, all but one attitudinal affective node showed relatively high node strength, indicating that these nodes were central and therefore potentially important for the COVID-19 broad attitude network. More specifically, the nodes that were of relatively high and comparable strength were *Negative Affect* (2.11), *Worries Virus* (2.11) and *Worries Measures* (2.04). This suggests that the attitudinal affective nodes have many and/or strong relations with other nodes in the network.

3.1.2 Community stability and detection analysis

The clusters of nodes as indicated by the community detection results from the communities in the network. Similar to the main text, ^ indicates binary nodes. *Consideration of Future Consequences*, *Resilience* and *Coping* were measured only in the first survey.

Fig S2. Community stability (network based on all participants that completed the first survey)

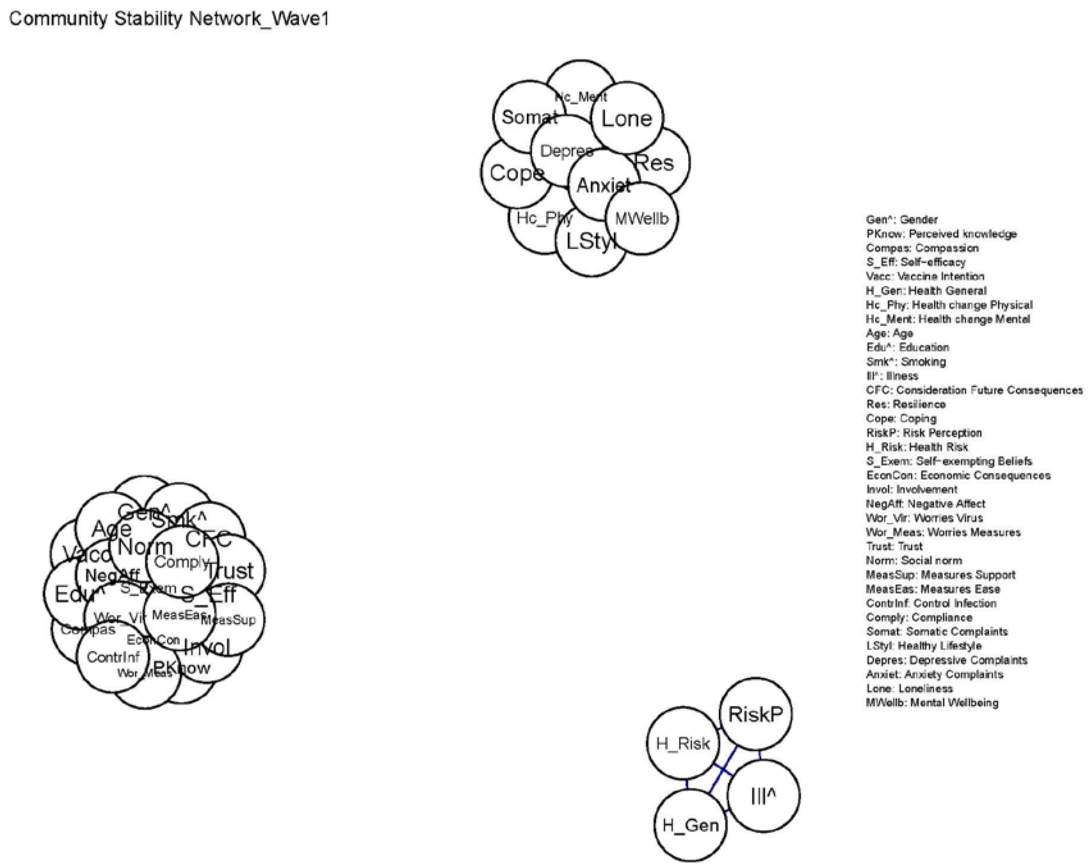

Fig S3. Community detection (network based on all participants that completed the first survey)

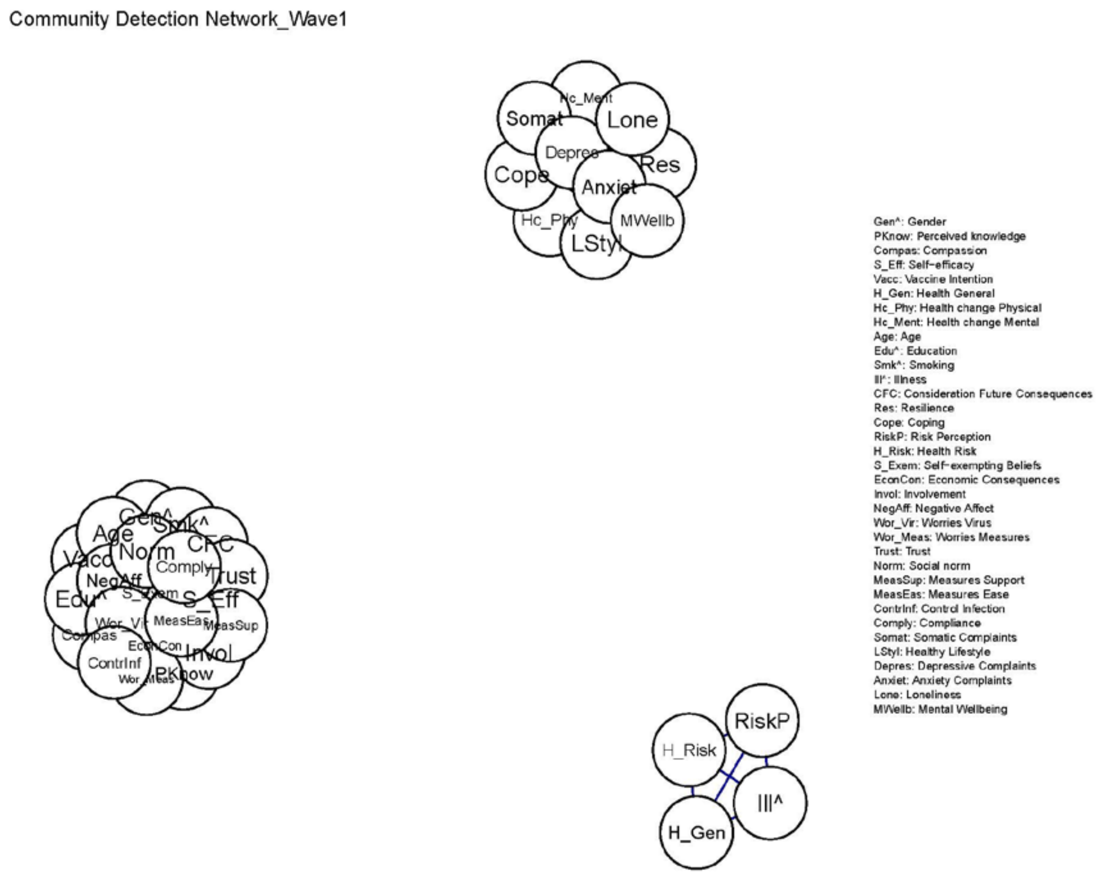

3.1.3 Network without threshold

Fig S4. Network wave 1. All edges are displayed (cutoff value .10: edges with weights below .10 are depicted with similar edge width and color density).

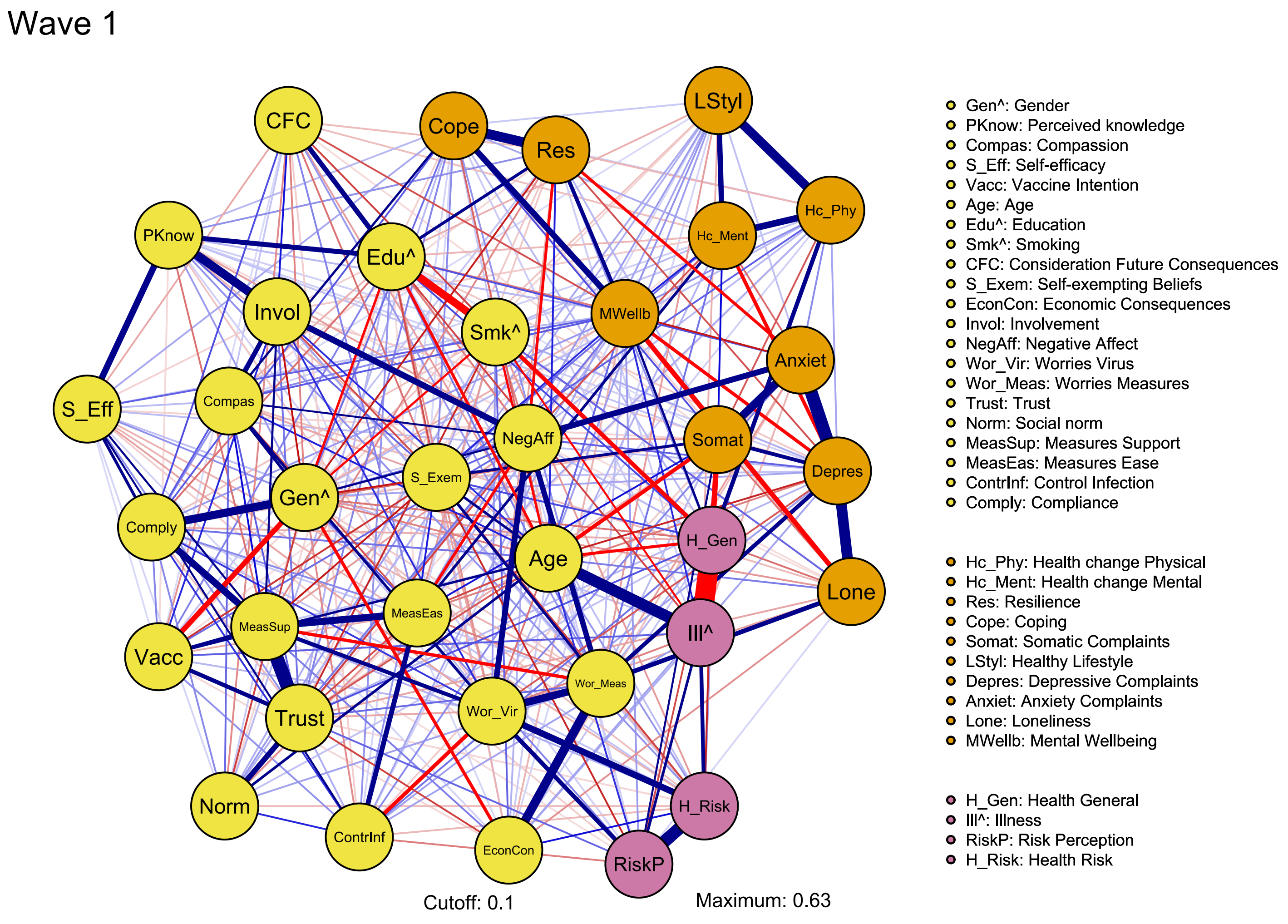

3.1.4 Edge weights

**Table S3. Total sample Network Wave 1**	**Gender**	**Perceived knowledge**	**Compassion**	**Self-efficacy**	**Vaccine Intention**	**Health General**	**Health change Physical**	**Health change Mental**	**Age**	**Education**	**Smoking**	**Illness**	**Consideration Future**	**Resilience**	**Coping**	**Risk Perception**	**Health Risk**	**Self-exempting Beliefs**	**Economic Consequences**	**Involvement**	**Negative Affect**	**Worries Virus**	**Worries Measures**	**Trust**	**Social norm**	**Measures Support**	**Measures Ease**	**Control Infection**	**Compliance**	**Somatic Complaints**	**Healthy Lifestyle**	**Depressive Complaints**	**Anxiety Complaints**	**Loneliness**	**Mental Wellbeing**
**Gender**		.04	.13	.04	-.20	-.06	.03		-.05	-.12	-.11		-.05	-.09	.06		-.02	-.09	-.14	-.10	.11	.02	-.02		-.08		.07	-.05	.28	-.06	-.02	.04	.06	-.04	-.03
**Perceived knowledge**				.23	-.02				-.03	.19	.02			.04	.03	.02	.04	.06		.30	-.04	-.02				-.06				.02		.02		-.01	.03
**Compassion**				.03	-.04			.02	.09	-.03	.05		.02	-.02	.11			-.04		.18	.10	.13		.06	.06	.10	-.07		.04		.02			-.03	.07
**Self-efficacy**					.01					-.02	.02	.03	-.03		.03	-.02	-.03		.02			-.04	-.03	.04	.02	.11		.10	.13	-.02		.03			.06
**Vaccine Intention**								-.01	.12	.05		.06	.01	.02		.06		-.08	-.02	.09	.06	.03		.17		.15	-.03	.04	.05		-.02				
**Health General**							.17		-.13	.07	-.17	-.63		.04	.03	-.07	-.09	.07	.02		.03		.03	.07	.02			.06	-.03	-.20				-.06	.12
**Health change Physical**								.24	.02		.02			-.03	.02			.06			.03				-.01		.03			-.07	.31	.04	.05		.04
**Health change Mental**										-.03	.03		-.03		.03			.06		-.02	-.07	.03	.06	-.01			.13			.10	.20	-.15	-.06		.09
**Age**										-.13	-.11	.37	-.04	.09	-.06	.08	.04	.12	.02	.08	-.02	-.05		-.07	.12	.02	-.07	.05	.06	-.16	.04	-.09		.05	.06
**Education**											-.27	-.07	.19	.13	-.02		.03	-.07	-.03		-.05	-.04	.05	.03	-.02	-.02	-.01	.04	-.06	.01	-.03	-.02	.07	-.02	-.05
**Smoking**												-.10	-.08		.02	.02	-.04	.03	.06		-.05	.04	-.02	-.08	.03		-.04	.02	-.02			.03		.06	.03
**Illness**														.02	.02	.12	.16		.03			-.05						.04		.07	-.03		-.03		.08
**Consideration Future Consequences**														-.03				-.06		.09	-.05		.04		-.05		.05	-.02	.07	-.01					-.07
**Resilience**															.33			-.02			-.13	-.04		-.02		-.05				.03		-.07	-.13		.16
**Coping**																		.05					.04	.05	.03		.02	.02	.07	.07	.02			-.02	.22
**Risk Perception**																	.41	-.09			.02	.17				-.01	.05	-.05		.10				.02	.04
**Health Risk**																		-.07	.09	.02		.23	-.03	-.02	-.03	.03	.02	-.03	.02	.04					
**Self-exempting Beliefs**																			.02	-.07	.06	-.04	.13		.04	-.09	.09	.04	-.06	.13	.03	.02			.08
**Economic Consequences**																				.02	.03		.30	-.07					.05		.02	.04	-.03	-.04	-.03
**Involvement**																					.23	.08	.03	.06		.11			.06			-.02			
**Negative Affect**																						.21	.20	-.08		-.04	-.13		.02	-.04	.02	.06	.22	-.01	-.02
**Worries Virus**																							.26	-.03		.16	.03	-.16	.08			-.07	.05	-.04	
**Worries Measures**																								.06	.04	-.14	-.05		.02	.03	-.04	.17	-.04	.18	.03
**Trust**																									.20	.44	-.07	.03	-.03	-.03		.03		-.04	.04
**Social norm**																										.02	-.04	.09	.05		.03				.05
**Measures Support**																											.25		.24						
**Measures Ease**																												.19	.05	.09	.04	-.05			.03
**Control Infection**																													.08		.04	.04	-.02		.02
**Compliance**																														-.03					.04
**Somatic Complaints**																																.16	.28	-.05	
**Healthy Lifestyle**																																.02			.04
**Depressive Complaints**																																	.42	.36	-.15
**Anxiety Complaints**																																			-.09
**Loneliness**																																			-.19
**Mental Wellbeing**																																			

3.1.5 Node strength

Table S4. Node strength value for each node.

Node	Strength
**Gender**	2.18
**Perceived knowledge**	1.22
**Compassion**	1.42
**Self-efficacy**	1.09
**Vaccine Intention**	1.34
**Health General**	2.16
**Health change Physical**	1.16
**Health change Mental**	1.35
**Age**	2.45
**Education**	1.99
**Smoking**	1.58
**Illness**	1.93
**Consideration Future Consequences**	0.98
**Resilience**	1.49
**Coping**	1.35
**Risk Perception**	1.35
**Health Risk**	1.50
**Self-exempting Beliefs**	1.88
**Economic Consequences**	1.09
**Involvement**	1.56
**Negative Affect**	2.11
**Worries Virus**	2.11
**Worries Measures**	2.04
**Trust**	1.83
**Social norm**	1.02
**Measures Support**	2.05
**Measures Ease**	1.70
**Control Infection**	1.21
**Compliance**	1.64
**Somatic Complaints**	1.80
**Healthy Lifestyle**	0.97
**Depressive Complaints**	2.07
**Anxiety Complaints**	1.53
**Loneliness**	1.22
**Mental Wellbeing**	1.95

3.1.6 Edge accuracy, Edge difference, Centrality stability, and Centrality difference

See OSF (https://osf.io/bhuvw/) for PDF's containing the output on edge accuracy, edge difference, centrality stability and centrality difference.

Output on edge accuracy and centrality stability is also displayed on the next pages. The edge accuracy plot is displayed to give a general impression of the width of the edge weight confidence intervals. Readers are referred to OSF for detailed information of specific edges.

Method

These analyses are conducted with bootstrap network estimation. In doing so, we specified the method to use as mgm (i.e., package bootnet, function bootnet, argument default = "mgm").

Interpretation guidance difference tests (see OSF)

Edge difference: a = .05, black boxes indicate significant differences between edges. Centrality difference: a = .05, black boxes indicate significant differences between nodes, node strength is presented in the diagonal boxes.

Fig S5. Edge accuracy (see OSF for PDF)

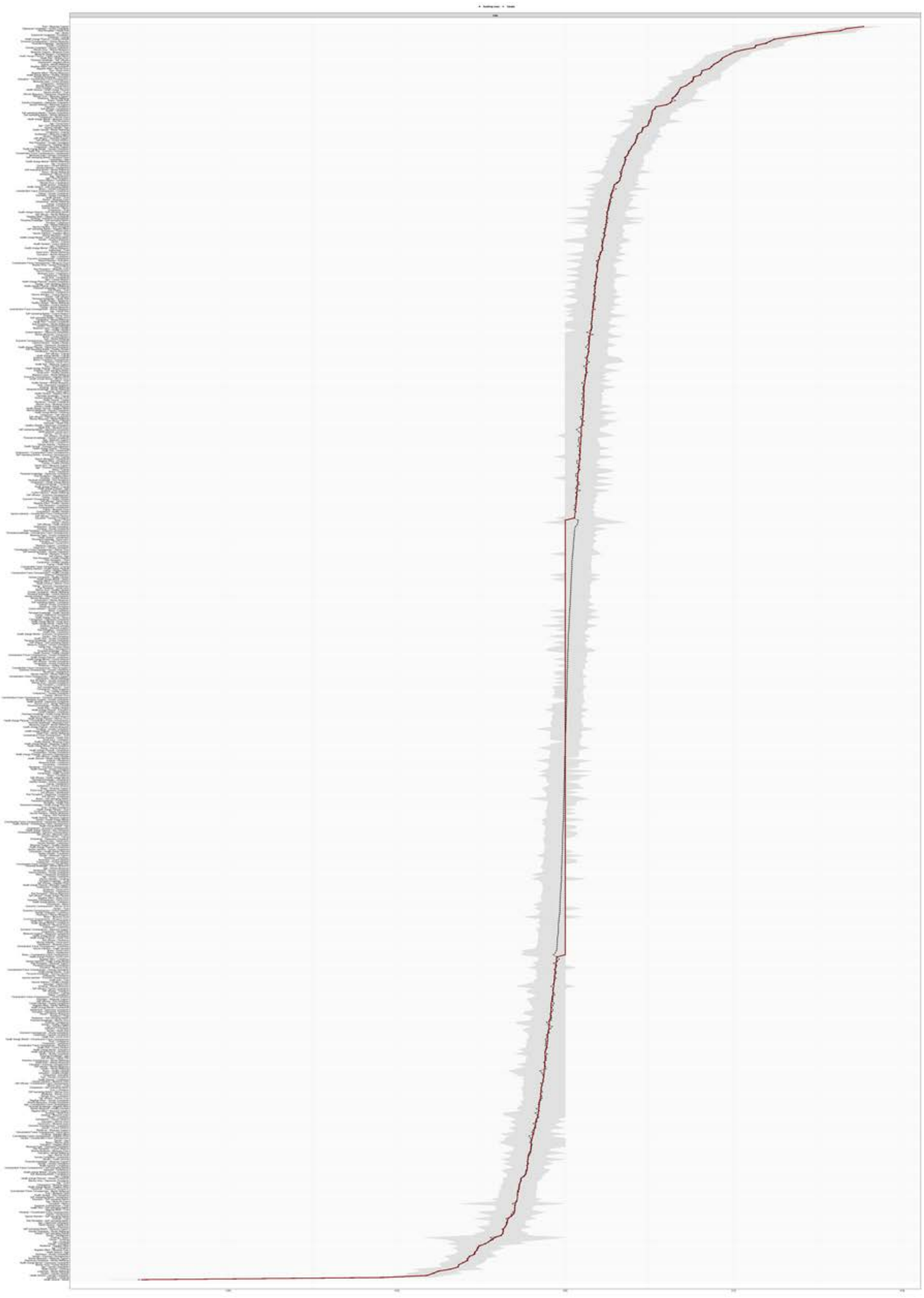

Fig S6. Centrality stability (See OSF for PDF)

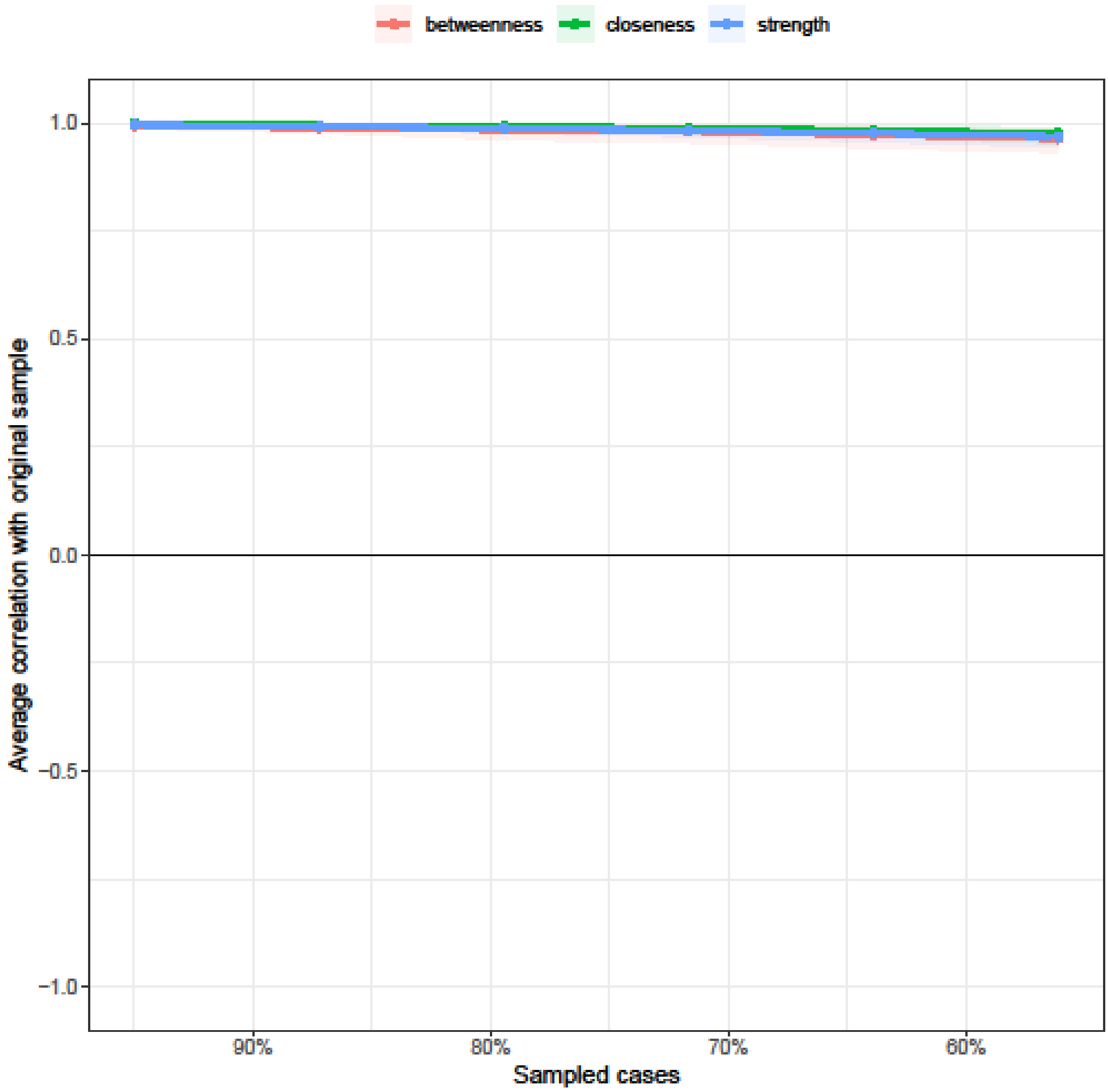

3.2 Part 2 - Interventions

3.2.1 Descriptives intervention conditions

Statistics of the nodes of wave 3 and 5, dived into the intervention conditions. First table includes one way ANOVA results for variables Health Risk, Economic Consequences, Negative Affect, Worries Virus and Measures Ease. Second table includes Kruskal-Wallis results for the rest of the variables. Includes the 27 nodes that can be subject to change.

**Table S5**	**Wave 3 (Valid *n* = 2,845)**					**Wave 5 (Valid *n* = 2,123)**				
	Control T3	Trust Low	Trust High	Social norm Low	Social norm High	Control T5	Measures Support Low	Measures Support High	Economic Consequences Low	Economic Consequences High
**Nodes[1]**	*M (SD)*	*M (SD)*	*M (SD)*	*M (SD)*	*M (SD)*	*M (SD)*	*M (SD)*	*M (SD)*	*M (SD)*	*M (SD)*
**Compliance**	5.83 (0.93)	5.84 (0.87)	5.93 (0.83)	5.85 (0.93)	5.95 (0.84)	5.74 (0.94)	5.6 (0.99)	5.74 (1.02)	5.6 (1.08)	5.64 (0.94)
**Risk Perception**	16.9 (9.11)	16.99 (8.5)	16.81 (9.22)	17.09 (9.33)	16.78 (8.5)	16.16 (8.8)	16.71 (9.31)	16.51 (8.85)	15.45 (8.62)	16.83 (9.16)
**Health Risk**	4.12 (1.23)	4.19 (1.18)	4.13 (1.22)	4.13 (1.23)	4.13 (1.19)	4.17 (1.27)	4.14 (1.26)	4.03 (1.3)	4.09 (1.2)	4.15 (1.25)
**Economic Consequences**	3.67 (1.34)	3.7 (1.38)	3.66 (1.37)	3.75 (1.35)	3.7 (1.32)	3.44 (1.33)	3.43 (1.35)	3.46 (1.42)	3.35 (1.19)	3.59 (1.29)
**Self-exempting Beliefs**	2 (1.13)	1.94 (1.1)	1.92 (1.09)	1.94 (1.11)	1.89 (0.98)	1.9 (1.09)	1.74 (0.95)	1.91 (1.11)	1.89 (1.06)	1.97 (1.08)
**Negative Affect**	3.45 (1.41)	3.42 (1.3)	3.36 (1.36)	3.43 (1.39)	3.39 (1.31)	3.07 (1.38)	3.07 (1.45)	3.11 (1.43)	2.98 (1.39)	3.28 (1.45)*
**Compassion**	4.77 (1.5)	4.74 (1.45)	4.82 (1.43)	4.74 (1.51)	4.63 (1.45)	4.56 (1.56)	4.4 (1.52)	4.57 (1.53)	4.47 (1.49)	4.55 (1.52)
**Worries Virus**	3.96 (1.22)	3.88 (1.18)	3.92 (1.28)	3.94 (1.22)	3.9 (1.27)	3.77 (1.28)	3.71 (1.25)	3.75 (1.25)	3.6 (1.16)	3.83 (1.25)
**Worries Measures**	3 (1.07)	3.03 (1.02)	2.99 (1.03)	3.07 (1.03)	3.03 (1.03)	2.81 (0.96)	2.81 (1.04)	2.81 (1.03)	2.7 (0.94)	2.97 (1.08)
**Vaccination Intention**	5.45 (1.92)	5.47 (1.86)	5.48 (1.86)	5.46 (1.88)	5.62 (1.86)	5.54 (1.91)	5.42 (1.91)	5.47 (1.91)	5.29 (1.95)	5.24 (2.01)
**Measures Support**	5.47 (1.17)	5.32 (1.23)	5.58 (1.15)	5.41 (1.21)	5.49 (1.15)	5.5 (1.2)	5.33 (1.23)	5.59 (1.23)	5.39 (1.28)	5.26 (1.32)
**Measures Ease**	3.83 (1.29)	3.81 (1.3)	3.92 (1.29)	3.87 (1.3)	3.79 (1.28)	4.04 (1.28)	3.83 (1.33)	4.03 (1.33)	3.84 (1.37)	3.81 (1.29)
**Social Norm**	4.68 (1.21)	4.57 (1.15)	4.87 (1.09)	4.42 (1.22)	5.02 (1.14)	4.44 (1.22)	4.16 (1.27)	4.48 (1.31)	4.32 (1.32)	4.35 (1.22)
**Control Infection**	4.43 (1.03)	4.4 (0.99)	4.54 (1.05)	4.53 (1.06)	4.5 (1.03)	4.62 (1.05)	4.56 (0.99)	4.61 (1.11)	4.52 (1.04)	4.49 (1.08)
**Self-efficacy**	5.52 (1.14)	5.58 (1.12)	5.61 (1.1)	5.59 (1.15)	5.62 (1.22)	5.67 (0.99)	5.52 (1.12)	5.69 (1.1)	5.59 (1.15)	5.52 (1.15)
**Involvement**	4.73 (1.29)	4.64 (1.24)	4.7 (1.22)	4.69 (1.27)	4.66 (1.27)	4.57 (1.33)	4.46 (1.29)	4.68 (1.21)	4.48 (1.32)	4.57 (1.27)
**Perceived Knowledge**	4.76 (1.13)	4.74 (1.08)	4.79 (1.15)	4.73 (1.17)	4.74 (1.14)	4.9 (1.14)	4.8 (1.18)	4.81 (1.14)	4.84 (1.15)	4.76 (1.15)
**Trust**	5.43 (1.19)	5.31 (1.1)	5.7 (1.06)	5.45 (1.1)	5.54 (1.1)	5.6 (1.14)	5.48 (1.14)	5.64 (1.11)	5.49 (1.15)	5.44 (1.18)
**Health General**	5.28 (1.23)	5.15 (1.31)	5.37 (1.17)	5.3 (1.23)	5.22 (1.27)	5.22 (1.2)	5.25 (1.24)	5.24 (1.24)	5.17 (1.34)	5.24 (1.23)
**Health change Physical**	4.01 (0.79)	4 (0.89)	4.02 (0.84)	4 (0.84)	3.98 (0.87)	3.98 (0.79)	3.99 (0.87)	4.03 (0.83)	3.88 (0.78)	4 (0.83)
**Health change Mental**	3.87 (0.87)	3.89 (0.9)	3.9 (0.9)	3.83 (0.86)	3.83 (0.88)	3.96 (0.86)	3.92 (0.83)	3.94 (0.95)	3.9 (0.83)	3.91 (0.84)
**Healthy Lifestyle**	3.87 (0.75)	3.86 (0.75)	3.83 (0.78)	3.88 (0.7)	3.84 (0.73)	3.88 (0.69)	3.88 (0.62)	3.92 (0.72)	3.86 (0.63)	3.91 (0.66)
**Mental Wellbeing~**	24.52 (4.2)	24.4 (4.17)	24.9 (4.37)	24.62 (4.54)	24.51 (4.02)	25.49 (4.63)	24.96 (4.39)	25.33 (4.85)	25.26 (4.48)	25.01 (4.48)
**Loneliness~**	2.27 (0.79)	2.25 (0.79)	2.22 (0.79)	2.28 (0.83)	2.3 (0.78)	2.11 (0.82)	2.22 (0.81)	2.12 (0.83)	2.19 (0.83)	2.21 (0.79)
**Somatic Complaints~**	1.32 (0.54)	1.35 (0.55)	1.33 (0.55)	1.32 (0.53)	1.31 (0.5)	1.28 (0.5)	1.26 (0.51)	1.29 (0.53)	1.3 (0.53)	1.33 (0.58)
**Depressive Complaints~**	1.85 (0.93)	1.83 (0.89)	1.79 (0.94)	1.83 (0.97)	1.86 (0.9)	1.65 (0.86)	1.67 (0.87)	1.7 (0.96)	1.67 (0.88)	1.74 (0.91)
**Anxiety Complaints~**	1.73 (0.88)	1.66 (0.8)	1.67 (0.85)	1.72 (0.92)	1.71 (0.81)	1.53 (0.76)	1.56 (0.82)	1.61 (0.88)	1.55 (0.8)	1.62 (0.85)

[1] 7-point Likert-scale, unless marked ~, indicating a 5-point Likert-scale. Please note that *Risk Perception* is the product of two items and *Mental Well-being* is the metric sum score of all items.

* p <.01; indicating a significant difference between the low, high and/or control condition of the intervention. A specification of these differences is provided in the manuscript.

Table S6	Wave 3 (Valid *n* = 2,845)					Wave 5 (Valid *n* = 2,123)				
	Control T3	Trust Low	Trust High	Social norm Low	Social norm High	Control T5	Measures Support Low	Measures Support High	Economic Consequences Low	Economic Consequences High
**Nodes**[1]	*Mdn (IQR)*	*Mdn (IQR)*	*Mdn (IQR)*	*Mdn (IQR)*	*Mdn (IQR)*	*Mdn (IQR)*	*Mdn (IQR)*	*Mdn (IQR)*	*Mdn (IQR)*	*Mdn (IQR)*
**Compliance**	6 (1)	6 (1.17)	6 (1)	6 (1.08)	6.17 (1)	5.83 (1.33)	5.83 (1.33)	5.83 (1.17)	5.83 (1.33)	5.83 (1.17)
**Risk Perception**	16 (14)	16 (14)	16 (14)	16 (14)	16 (14)	15 (15)	16 (15)	16 (14.75)	15 (12)	16 (14)
**Health Risk**	4 (1.5)	4 (1.5)	4 (1.5)	4 (1.5)	4 (1.5)	4 (1.75)	4 (1.5)	4 (2)	4 (2)	4 (1.5)
**Economic Consequences**	4 (2)	3.5 (2.5)	3.5 (2)	4 (2)	3.5 (1.5)	3.5 (2)	3 (2)	3.5 (2)	3.5 (1.5)	3.5 (2)
**Self-exempting Beliefs**	1.5 (1.5)	1.5 (1.5)	1.5 (1.5)	1.5 (1.5)	1.5 (1.5)	1.5 (1.5)	1.5 (1.5)	1.5 (1.5)	1.5 (1.5)	1.5 (1.5)
**Negative Affect**	3.5 (2)	3.38 (1.88)	3.25 (2)	3.38 (2)	3.5 (2)	3 (2)	3 (2.38)	3 (2.13)	2.88 (1.88)	3.25 (2.25)
**Compassion**	5 (2)	5 (2)	5 (2)	5 (2)	5 (2)	5 (2)	5 (1)	5 (2)	5 (2)	5 (2)
**Worries Virus**	4 (1.5)	4 (1.75)	4 (1.75)	4 (1.5)	4 (1.75)	3.75 (2)	3.75 (1.75)	3.75 (1.75)	3.5 (1.5)	3.75 (1.75)
**Worries Measures**	3 (1.5)	3 (1.33)	3 (1.17)	3 (1.33)	3 (1.33)	2.83 (1.17)	2.67 (1.5)	2.67 (1.5)	2.67 (1.25)	2.83 (1.5)*
**Vaccination Intention**	6 (3)	6 (3)	6 (3)	6 (3)	7 (2)	7 (3)	6 (3)	6 (3)	6 (3)	6 (3)
**Measures Support**	5.71 (1.57)	5.57 (1.71)	5.86 (1.43)*	5.71 (1.57)	5.71 (1.57)	5.71 (1.57)	5.71 (1.43)	5.86 (1.57)*	5.71 (1.57)	5.57 (1.57)
**Measures Ease**	4 (1.5)	4 (1.5)	4 (2)	4 (1.5)	4 (1.5)	4 (2)	4 (1.5)	4 (2)	4 (1.75)	4 (1.5)
**Social Norm**	5 (1.5)	4.5 (1.5)	5 (1.5)**	4.5 (2)	5 (1.5)**	4.5 (1.5)	4 (1.5)	4.5 (2)*	4.5 (1.5)	4.5 (1.5)
**Control Infection**	4.5 (1)	4.5 (1)	4.5 (1)	4.5 (1)	4.5 (1)	4.5 (1)	4.5 (1)	4.5 (1.5)	4.5 (1)	4.5 (1)
**Self-efficacy**	6 (1)	6 (1)	6 (1)	6 (1)	6 (1)	6 (1)	6 (1)	6 (1)	6 (1)	6 (1)
**Involvement**	5 (1.67)	4.67 (2)	4.67 (1.67)	5 (1.83)	4.67 (1.67)	4.67 (2)	4.33 (1.67)	4.67 (1.67)	4.67 (2)	4.67 (2)
**Perceived Knowledge**	5 (2)	5 (1)	5 (2)	5 (2)	5 (2)	5 (2)	5 (2)	5 (2)	5 (2)	5 (2)
**Trust**	5.75 (1.5)	5.5 (1.25)	6 (1)**	5.5 (1.5)	5.75 (1.25)	6 (1.5)	5.75 (1.31)	6 (1.25)	5.75 (1.25)	5.5 (1.5)
**Health General**	6 (1)	5 (1)	6 (1)	6 (1)	6 (1)	5 (1)	6 (1)	6 (1)	5 (1)	6 (1)
**Health change Physical**	4 (0)	4 (0)	4 (0)	4 (0)	4 (0)	4 (0)	4 (0)	4 (0)	4 (0)	4 (0)
**Health change Mental**	4 (0)	4 (0)	4 (0)	4 (0)	4 (1)	4 (0)	4 (0)	4 (0)	4 (0)	4 (0)
**Healthy Lifestyle**	4 (0.33)	4 (0.58)	4 (0.67)	4 (0.33)	4 (0.33)	4 (0.33)	4 (0.33)	4 (0.33)	4 (0.33)	4 (0.33)
**Mental Wellbeing~**	24.11 (5.49)	24.11 (5.49)	25.03 (6.59)	25.03 (5.49)	25.03 (5.49)	25.03 (6.96)	25.03 (5.78)	25.03 (5.78)	25.03 (5.78)	25.03 (5.78)
**Loneliness~**	2.17 (1.04)	2.17 (1)	2 (1)	2.17 (1.17)	2.17 (1.17)	2 (1)	2 (1)	2 (1.17)	2.17 (1.08)	2 (1)
**Somatic Complaints~**	1 (0.5)	1.17 (0.5)	1 (0.5)	1 (0.5)	1 (0.33)	1 (0.33)	1 (0.33)	1 (0.33)	1 (0.33)	1 (0.5)
**Depressive Complaints~**	1.5 (1.33)	1.5 (1.33)	1.33 (1.17)	1.5 (1.33)	1.5 (1.17)	1.33 (1)	1.33 (1)	1.17 (1.13)	1.17 (1.17)	1.33 (1.17)
**Anxiety Complaints~**	1.33 (1.17)	1.33 (1)	1.33 (1)	1.33 (1)	1.33 (1.17)	1.17 (0.83)	1.17 (0.83)	1.17 (0.83)	1.17 (0.92)	1.17 (1)

[1] 7-point Likert-scale, unless marked ~, indicating a 5-point Likert-scale. Please note that *Risk Perception* is the product of two items and *Mental Well-being* is the metric sum score of all items.

* p <.01; ** p <.001; indicating a significant difference between the low, high and/or control condition of the intervention. A specification of these differences is provided in the manuscript.

3.2.2 Significance values of difference tests per node

Table S7. Significance values (p-values) per node for each intervention for comparing conditions (including control condition).

Test	Node	Interventions			
		T3 Trust	T3 Social Norm	T5 Measures Support	T5 Consequences Economy
		p-value	*p*-value	*p*-value	*p*-value
**Kruskal-Wallis**	Compliance	.157	.131	.027	.161
	Risk Perception	.741	.959	.743	.143
	Self-exempting beliefs	.413	.427	.073	.358
	Compassion	.656	.247	.196	.514
	Worries Measures	.692	.518	.897	.001*
	Vaccination Intention	.953	.169	.392	.017
	Measures Support	.001*	.476	.001*	.017
	Social Norm	.000*	.000*	.001*	.315
	Perceived Control	.161	.330	.513	.193
	Self-efficacy	.347	.097	.044	.226
	Involvement	.320	.502	.072	.431
	Perceived Knowledge	.719	.934	.316	.279
	Trust	.000*	.191	.054	.069
	Health General	.053	.683	.859	.844
	Health change Physical	.976	.814	.424	.077
	Health change Mental	.904	.797	.861	.954
	Healthy Lifestyle	.771	.693	.429	.608
	Mental Well-being	.144	.714	.252	.432
	Loneliness	.353	.731	.040	.070
	Somatic Complaints	.336	.918	.419	.548
	Depression Complaints	.272	.396	.951	.199
	Anxiety Complaints	.496	.655	.772	.307
**ANOVA**	Health Risk	.602	.980	.202	.606
	Consequences Economy	.885	.493	.952	.018
	Negative Affect	.496	.786	.897	.007*
	Worries Virus	.530	.738	.802	.024
	Measures Ease	.332	.643	.048	.021

* $p < .01$; indicating a significant difference between the low, high and/or control condition of the intervention. A specification of these differences is provided in the manuscript.

4. References

1. Bish A, Michie S. Demographic and attitudinal determinants of protective behaviours during a pandemic: A review. British Journal of Health Psychology. 2010;15(4):797-824.

2. Ajzen I. The Theory of Planned Behavior. Organizational Behavior and the Human Decision Process. 1991;50:179-211.

3. Rosenstock IM, Strecher VJ, Becker MH. Social Learning Theory and the Health Belief Model. Health Education Quarterly. 1988;15(2):175-83.

4. Rosenstock IM. Historical Origins of the Health Belief Model. Health Education Monographs. 1974;2(4):328-35.

5. Bogg T, Milad E. Demographic, personality, and social cognition correlates of coronavirus guideline adherence in a U.S. sample. Health Psychology. 2020;39(12):1026–36.

6. van Rooij B, de Bruijn AL, Reinders Folmer C, Kooistra EB, Kuiper ME, Brownlee M, et al. Compliance with COVID-19 Mitigation Measures in the United States. 2020. Available from: https://doi.org/10.31234/osf.io/qymu3.

7. Shahnazi H, Ahmadi-Livani M, Pahlavanzadeh B, Rajabi A, Hamrah MS, Charkazi A. Assessing preventive health behaviors from COVID-19: a cross sectional study with health belief model in Golestan Province, Northern of Iran. Infectious Diseases of Poverty. 2020;9(6):91-9.

8. Li J, Yang A, Dou K, Wang L, Zhang M, Lin X. Chinese public's knowledge, perceived severity, and perceived controllability of COVID-19 and their associations with emotional and behavioural reactions, social participation, and precautionary behaviour: a national survey. BMC Public Health. 2020;20:1589.

9. Plohl N, Musil B. Modeling compliance with COVID-19 prevention guidelines: The critical role of trust in science. Psychology, Health & Medicine. 2021;26(1):1-12.

10. Siegrist M, Zingg A. The Role of Public Trust During Pandemics. European Psychologist. 2014;19(1):23-32.

11. Lin Y, Huang L, Nie S, Liu Z, Yu H, Yan W, et al. Knowledge, Attitudes and Practices (KAP) related to the Pandemic (H1N1) 2009 among Chinese General Population: a Telephone Survey. BMC Infectious Diseases. 2011;11(1):128.

12. de Zwart O, Veldhuijzen IK, Richardus JH, Brug J. Monitoring of risk perceptions and correlates of precautionary behaviour related to human avian influenza during 2006 - 2007 in the Netherlands: results of seven consecutive surveys. BMC Infect Dis. 2010;10:114.

13. Brouard S, Vasilopoulos P, Becher M, Brouard S, Vasilopoulos P, & , Becher M, et al. Sociodemographic and Psychological Correlates of Compliance with the COVID-19 Public Health Measures in France. Canadian Journal of Political Science Revue Canadienne De Science Politique,. 2020:1–6.

14. Carvalho LF, Pianowski G, Gonçalves AP. Personality differences and COVID-19: are extroversion and conscientiousness personality traits associated with engagement with containment measures? Trends in Psychiatry and Psychotherapy. 2020;42(2):179-84.

15. Leigh-Hunt N, Bagguley D, Bash K, Turner V, Turnbull S, Valtorta N, et al. An overview of systematic reviews on the public health consequences of social isolation and loneliness. Public Health. 2017;152:157-71.

16. Harper CA, Satchell LP, Fido D, Latzman RD. Functional Fear Predicts Public Health Compliance in the COVID-19 Pandemic. International Journal of Mental Health and Addiction. 2021;19; 1875-1888.

17. Chambon M, Dalege J, Borsboom D, Waldorp LJ, van der Maas HLJ, van Harreveld F. How compliance with behavioural measures during the initial phase of a pandemic develops over time: a longitudinal COVID-19 study. British Journal of Social Psychology. Forthcoming 2022.

18. van Borkulo CD, van Bork R, Boschloo L, Kossakowski JL, Tio P, Schoevers RA, et al. Comparing network structures on three aspects: A permutation test. Psychological Methods. Forthcoming 2022.

19. Government of the Netherlands. Dutch measures against coronavirus: basic rules for everyone 2020 [Available from: https://www.government.nl/topics/coronavirus-covid-19/tackling-new-coronavirus-in-the-netherlands/basic-rules-for-everyone.

20. National Institute for Public Health and the Environment. Measures to prevent the spread of COVID-19 2020 [Available from: https://www.rivm.nl/en/novel-coronavirus-covid-19.

21. Chambon M, Dalege J, Elberse JE, van Harreveld F. A Psychological Network Approach to Attitudes and Preventive Behaviors During Pandemics: A COVID-19 Study in the United Kingdom and the Netherlands. Social Psychological and Personality Science. 2022;13(1):233-45.

22. Wolff K, Larsen S, Øgaard T. How to define and measure risk perceptions. Annals of Tourism Research. 2019;79:102759.

23. Strathman A, Gleicher F, Boninger D, Edwards S. The Consideration of Future Consequences: Weighing Immediate and Distant Outcomes of Behavior. Journal of Personality and Social Psychology. 1994;66:742-52.

24. Smith BW, Dalen J, Wiggins K, Tooley E, Christopher P, Bernard J. The brief resilience scale: Assessing the ability to bounce back. International Journal of Behavioral Medicine. 2008;15(3):194-200.

25. Garnefski N, Kraaij V. Cognitive emotion regulation questionnaire – development of a short 18-item version (CERQ-short). Personality and Individual Differences. 2006;41(6):1045-53.

26. Kalisch R, Veer I, Yuen K, Hendler T, Myin-Germeys I, Walter H, et al. DynaCORE-C: The DynaMORE cross-sectional survey study on psychological resilience to the mental health consequences of the Corona crisis. 2020. Available from: osf.io/5xq9p

27. Tennant R, Hiller L, Fishwick R, Platt S, Joseph S, Weich S, et al. The Warwick-Edinburgh Mental Well-being Scale (WEMWBS): development and UK validation. Health and Quality of Life Outcomes. 2007;5(1):63.

28. de Jong Gierveld J, van Tilburg T. De ingekorte schaal voor algemene, emotionele en sociale eenzaamheid. Tijdschrift voor Gerontologie en Geriatrie. 2008;39(1):4-15.

29. Derogatis LR. BSI 18, Brief Symptom Inventory 18: Administration, scoring and procedures manual: NCS Pearson, Incorporated; 2001.

30. R Core Team. R: A language and environment for statistical computing Vienna, Austria: R Foundation for Statistical Computing; 2013 [Available from: http://www.R-project.org/.

31. Dalege J, Borsboom D, van Harreveld F, van der Maas HLJ. Network Analysis on Attitudes: A Brief Tutorial. Social Psychological and Personality Science. 2017;8(5):528-37.

32. Haslbeck JMB, Waldorp LJ. mgm: Estimating Time-Varying Mixed Graphical Models in High-Dimensional Data. Journal of Statistical Software. 2020;93(8):1-46.

33. Epskamp S, Cramer AOJ, Waldorp LJ, Schmittmann VD, Borsboom D. qgraph: Network Visualizations of Relationships in Psychometric Data. Journal of Statistical Software. 2012;48(4):1-18.

34. Epskamp S, Borsboom D, Fried EI. Estimating psychological networks and their accuracy: A tutorial paper. Behavior Research Methods. 2018;50(1):195-212.

35. Csardi G, Nepusz T. The igraph software package for complex network research. InterJournal. 2006;Complex Systems:1695.
