# Supplementary material for: Tailored interventions into broad attitude networks towards the COVID-19 pandemic
Source: PLoS One. 2022 Oct 27;17(10):e0276439. doi: 10.1371/journal.pone.0276439 (PMC9612523; doi:10.1371/journal.pone.0276439)
Supplement: Attachment — Submitted filename: ResponseToReviewers_PONE-D-22-06537R1.docx [file pone.0276439.s003.docx]

Subject: revision PONE-D-22-06537R1

Dear Dr. Spruyt,

Please find enclosed a revised version of our manuscript ‘Tailored interventions into broad attitude networks towards the COVID-19 pandemic’ (PONE-D-22-06537R1). We would like to thank you for the opportunity for an improvement of the manuscript. We will address each issue more specifically below.

Comments by reviewers

**1) Should Zeal and Neal (2021) be cited when mentioning criticism of centrality, specifically re: the boundary specification problem (doi: 10.1037/met0000426). If this paper is covered in the cited Hallquist paper cited then perhaps not, but otherwise I think Zeal probably should be cited here.**

We agree that this paper is relevant and included the citation in the revised manuscript.

**2) Should the IQR range be added to Table 3 for the medians?**

We are thankful for this suggestion and included the IQR in Table 3 in the revised manuscript.

**3) Regarding my initial comment about whether a nodes edge set may influence strength centrality, I’m not sure the authors reply did completely address this. I understand that edges in an MGM all have the same substantive interpretation e.g. conditional associations, but what I don’t think the reply did address (unless I’m mistaken), is what effect averaging different types of regression coefficients may have on calculating strength centrality. For example (if I understand the MGM correctly), if you have a continuous node connected to two other continuous nodes then edge weights are averaged linear regression coefficients and strength centrality is the sum of these two averages. But if you have a continuous node connected to one other continuous node as well as a binary node, then one edge is an average of two linear regression coefficients, and the other edge is an average of a linear regression coefficient and a logistic regression coefficient, and strength centrality is thus the sum of different types of coefficient averages compared to the first node. Is it valid to then compare the two nodes on strength centrality? (even if the edges themselves can all still be interpreted as conditional associations)? It is however quite possible I am misunderstanding, because I am not an expert in psychometrics or statistics.**

This is indeed an interesting and very relevant question. In general your intuition is correct, that parameters obtained from random variables with all kinds of distributions are difficult to compare. For our specific situation though, parameters are comparable. We will try and explain this better.

You could think of the following situation. Suppose we have a Gaussian random variable Y with two predictors X and Z, and X is Gaussian and Z is binary. Then changing X one unit (on a continuous scale) results in a change in Y, and this is similar to changing Z one unit (on a binary scale) resulting in a change in Y. The coefficients for X and Z are then comparable because X and Z change Y in a similar way and X and Z are exponential family random variables. So, in general such comparisons of edge parameters is not possible. But in our case they are.

We hope this answers the question.

We are again thankful for the comments made by the reviewers. Hopefully, we have adequately dealt with all of them and we are looking forward to hearing from you.
